# Genetic diversity of *Venturia carpophila* populations from different hosts and geographic regions in China

**DOI:** 10.3389/fmicb.2022.985691

**Published:** 2022-12-16

**Authors:** Yang Zhou, Chingchai Chaisiri, Mei Luo, Fei Fan, Yu-Fu Wang, Liang-Fen Yin, Wei-Xiao Yin, Chao-Xi Luo

**Affiliations:** ^1^Oil Crops Research Institute of the Chinese Academy of Agricultural Sciences, Wuhan, China; ^2^Key Lab of Horticultural Plant Biology, Ministry of Education, Huazhong Agricultural University, Wuhan, China; ^3^College of Plant Science and Technology, Huazhong Agricultural University, Wuhan, China; ^4^Experimental Teaching Center of Crop Science, Huazhong Agricultural University, Wuhan, China; ^5^Hubei Key Laboratory of Plant Pathology, Huazhong Agricultural University, Wuhan, China

**Keywords:** peach, *Venturia carpophila*, genetic diversity, populations, specificity

## Abstract

*Venturia carpophila*, the causal agent of scab disease of peach, mume, and apricot, is widely distributed around the world. Scab of stone fruits is an important disease in China. However, little is known about the population biology and genetic diversity of the *V. carpophila*. To better understand the genetic diversity and population structure of *V. carpophila*, 186 single-spore isolates from different hosts and geographic regions were obtained and analyzed by using 31 simple sequence repeat (SSR) markers. This included 156 isolates from peach spanning 14 provinces, 15 isolates from mume and 15 isolates from apricot in Huazhong Agricultural University (HZAU). Diversity analysis with SSR markers showed a low incidence of polymorphisms within mume isolates (32.59% of markers), but a higher incidence of polymorphisms within peach isolates (42.96%) and apricot isolates (57.04%). Within peach isolates, Nei’s average gene diversity ranged from 0.07 for Hebei population to 0.18 for Hubei population. AMOVA analysis revealed that 13% of the observed genetic diversity was partitioned among the geographic populations, while 40% of the observed genetic diversity was partitioned among the host populations. Other analyses (PCoA, STRUCTURE, DAPC, MSN, and UPGMA) indicated that the Chinese *V. carpophila* populations could be clustered into three distinct genetic groups, which correspond to the host boundaries of peach, mume and apricot. The genetic identity of *V. carpophila* isolates throughout the range is dependent on hosts, but not geographic regions.

## Introduction

**Figure 1:**
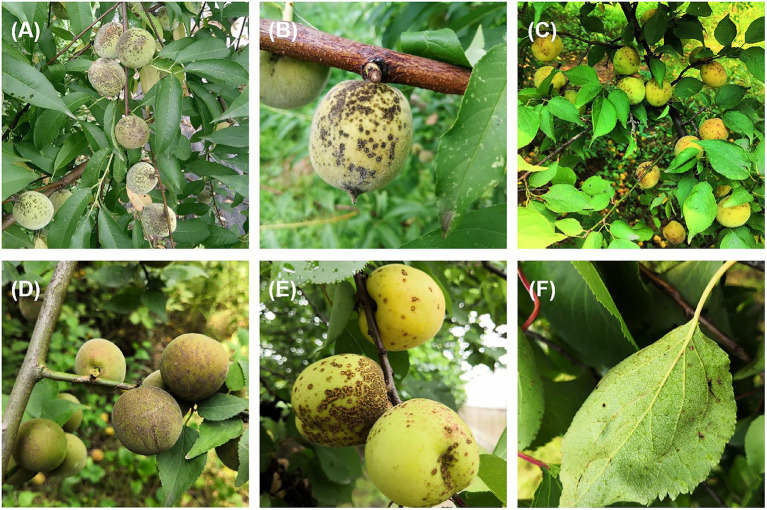
Typical symptoms of scab on peach, mume, and apricot. **(A,B)** Typical symptoms on peach, **(C,D)** on mume, **(E,F)** on apricot fruit and leaf, respectively.

Stone fruit trees such as peach (*Prunus persica*), mume (*P. mume*), and apricot (*P. armeniaca*) are grown widely in China (Chuda et al., 1999; Li et al., 2018). *Venturia carpophila* is an important pathogen that causes scab disease on several stone fruits including peach, mume, apricot, and almond (*Prunus dulcis*; Sivanesan, 1977; Chen et al., 2014; Bailly, 2020). The scab disease can result in yield loss, fruit quality decline, and increased management costs. Scab is one of the most serious diseases on peach in China, which can cause defoliation and tree weakening, and more susceptible to chilling and freezing injuries (Zhou et al., 2021a). Typical symptoms on fruits are black freckles, spots, and/or scabs of variable sizes, distribution and density, irregularly circular to oval lesions, developing a slightly raised dark-brown purple border and be slightly raised and corky in appearance (Scherm et al., 2008; Chen et al., 2017; Kim et al., 2017; Figure 1). Multiple spots on fruits often merge, leading to cracking of the skin (Figures 1A,B,E). Leaf symptoms are less common, but occur as yellowish blotches and then turn grayish as spores are produced (Chen et al., 2014; Figure 1F). In our experiments, we found that isolates from three hosts showed obvious differences in colony morphology, growth rate and spore production on the MEA medium. On MEA medium after 42 days at 21°C, isolates from apricot were light gray in color and were fast growing with approximately 3 cm colonies in diameter and the least conidia production (0.9 × 10^4^/dish). Isolates from mume were dark gray in color and the fastest growing with approximately 3.3 cm colonies in diameter and better conidia production (2 × 10^4^/dish). Isolates from peach were white to gray in color and were slow growing with approximately 2.6 cm colonies in diameter and the best conidia production (10 × 10^4^/dish, unpublished data). Conidia of the pathogens are spread by rain splash and wind. The disease is particularly severe in temperate climate regions characterized by humid and cool springs, and poor air circulation (Bock et al., 2020). If the weather condition is favorable, asexual conidia release and cause massive secondary infections during the growing season (Villarino et al., 2012; González-Domínguez et al., 2017). Scabs can result in fruit downgrading and/or rejection if the infection is severe, and these blemishes reduce the value of fruit intended for the fresh market and cause huge economic losses (Schnabel and Layne, 2004). During the sample collection, the infection could be observed in up to 100% of fruits in some orchards (Figures 1A,C). Control of scabs is mainly based on the application of fungicides in China. So far, carbendazim resistance has been detected (Zhou et al., 2021a). Integrated management approaches such as planting resistant cultivars could potentially reduce scab inocula in orchards.

With the development of modern molecular biology, molecular markers have become important tools to study the genetic diversity and population structure of pathogenic fungi. Different molecular tools have been used to characterize the genetic diversity of populations of *Venturia* spp., including random amplified polymorphic DNA (RAPD; Koh et al., 2013; Chen et al., 2014; Poniatowska et al., 2016), restriction fragment length polymorphism (RFLP; Tenzer and Gessler, 1999; Guérin et al., 2004; Padder et al., 2013), amplified fragment length polymorphism (AFLP; Gladieux et al., 2008), and simple sequence repeat marker (SSR; Tenzer and Gessler, 1999; Guérin et al., 2004; Chen et al., 2018). SSR markers have the advantages of high polymorphisms, stability, codominant inheritance, and easy operation. At present, SSR technology has been widely used in variety identification, genetic classification, mapping, core germplasm identification and evaluation, and genetic diversity research of pathogenic fungi, including *Verticillium dahliae* (Jiménez-Díaz et al., 2011; Baroudy et al., 2019), *V. inaequalis* (Tenzer and Gessler, 1999; Leroy et al., 2013; Ebrahimi et al., 2016), *V. nashicola* (Choi et al., 2019), *Monilinia fructicola* (Fan et al., 2010; Villarino et al., 2012), and *Ustilaginoidea virens* (Sun et al., 2013; Yu et al., 2016). In this study, SSR markers were used to make a fundamental and thorough analysis of the genetic diversity and population structure of *V. carpophila*. Although the genetic diversity was carried out on *V. inaequalis* from different regions of the world, the characterization of *V. carpophila* has rarely been reported, most likely the latter one is very difficult to be obtained in the laboratory. Two molecular tools have been used to characterize the genetic diversity of populations of *V. carpophila* in the United States. Results revealed some divergence between two different geographic *V. carpophila* populations, and different levels of genetic diversity were observed within the two populations (Chen et al., 2014, 2018).

Stone fruit scab is widely distributed in China, yet there is no information about the genetic diversity of Chinese *V. carpophila* populations. In this study, we collected samples and isolated single-spore isolates from three hosts and 14 provinces in China. Population genetics analysis is crucial for understanding the population genetic structure and diversity, thus helping to prevent and control the disease (Sitther et al., 2018; Choi et al., 2019; He et al., 2019; Singh and Khan, 2019). Considering that scab disease is one of the most important peach diseases in China, the population structure needs to be studied and monitored. The objectives of this study are to determine (i) if all scab-like symptoms on different stone fruits (peach, mume, and apricot) are caused by *V. carpophila*, (ii) the genetic diversity and population structure of *V. carpophila* in China, and (iii) if the genetic differences are specifically correlated with geographic regions or hosts.

## Materials and methods

### Isolation of *Venturia carpophila*

**Figure 2:**
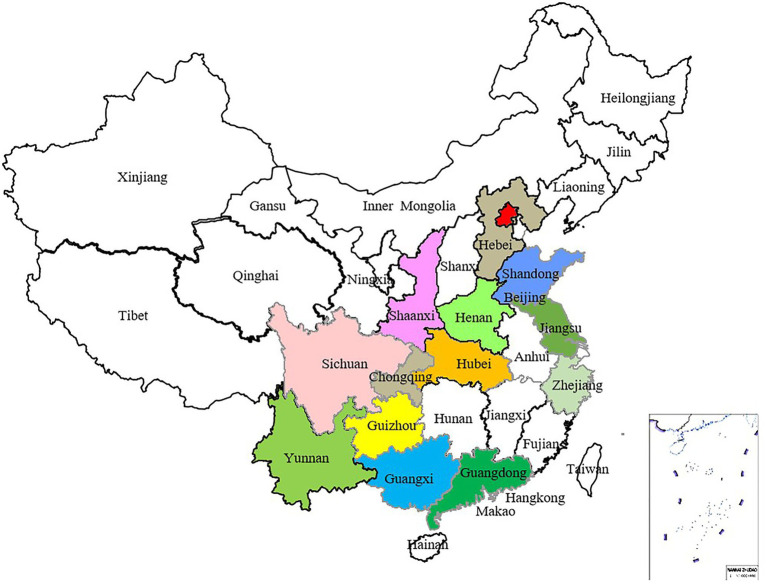
Distribution map of 186 *Venturia carpophila* isolates, which were collected from 14 major peach-producing provinces in China.

From 2017 to 2019, samples of stone fruit scab were collected from 14 provinces in China, among them, peach scab samples were collected from 14 provinces, and all the apricot scab and mume scab samples were collected from Hubei Province (Table 1). Altogether 750 isolates were obtained using the single spore isolation method, from which 186 isolates were selected for genetic diversity analysis (Table 1; Figure 2). Among selected isolates, 156 were from peach or nectarine that were located in 14 provinces in China, 15 were from mume and 15 were from apricot in Huazhong Agricultural University (HZAU; Table 1), the isolates from mume and apricot are mainly used as references. Briefly, asexually produced conidia of *V. carpophila* were scraped from a single lesion on the fruit surface using a scalpel and then spread with a scalpel on the surface of water agar (20 g of agar per liter) plates. Using a modified microscope (Wuhan Heipu Science and Technology Ltd., Wuhan, China), a single spore was isolated with a glass needle and then transferred to potato dextrose agar (PDA; 200 g of potato, 20 g of dextrose, and 20 g of agar per liter) amended with lactic acid (0.50 ml/L), streptomycin sulfate (0.20 g/L), tetracycline (0.05 g/L), and chloramphenicol (0.05 g/L; Chen et al., 2014). For long-term storage, isolates were maintained on dried filter paper. In brief, colonies were grown on antibiotic-amended oatmeal agar medium (OMA; 30 g of oatmeal; 20 g of agar per liter) in petri dishes starting with mycelial suspension, and sterile filter paper discs (5 mm in diameter) were placed on the OMA surface (Zhou et al., 2020, 2022). Each isolate was grown at 21°C for about 14 days in darkness. When the filter discs were fully covered with mycelium, they were removed and dried in a desiccator with silica gel. After 10 days, discs were transferred into 1.5-ml sterile centrifuge tubes with desiccated silica gel and stored at −20°C (Chen et al., 2019; Zhou et al., 2021b).

**Table 1:** Sampling geographic regions, host, cultivar, and tissue of isolates of *Venturia carpophila* collected to assess population genetic diversity.

Isolate	Host	Origin	Tissue	rDNA-ITS^a^	*LSU*
BJ1-1-1	Peach	Beijing	Fruit	NA	NA
BJ1-3-1	Peach	Beijing	Fruit	NA	NA
BJ2-3-1	Nectarine	Beijing	Fruit	MN958548	MT845672
BJ2-4-2	Nectarine	Beijing	Fruit	MN958549	MT845673
BJ2-7-1	Peach	Beijing	Fruit	NA	NA
BJ2-8-1	Nectarine	Beijing	Fruit	MN958550	MT845674
BJ3-4-1	Nectarine	Beijing	Fruit	NA	NA
CQBB1-2-1	Peach	Beibei, Chongqing	Fruit	NA	NA
CQBN1-1-1-3	Peach	Hechuan, Chongqing	Fruit	MN958551	MT845675
CQHC1-12–1-1	Peach	Hechuan, Chongqing	Fruit	MN958552	MT845676
CQHC1-3-1-1	Peach	Hechuan, Chongqing	Fruit	NA	NA
CQHC2-4-1-2	Peach	Hechuan, Chongqing	Fruit	NA	NA
CQHC2-4-1-4	Peach	Hechuan, Chongqing	Fruit	NA	NA
CQHC2-4-1-5	Peach	Hechuan, Chongqing	Fruit	NA	NA
CQSPB3-3	Peach	Shapinba, Chongqing	Fruit	MN958553	MT845677
CQTN1-3-1-1	Peach	Tongnan, Chongqing	Fruit	MN958554	MT845678
CQTN1-3-2-2	Peach	Tongnan, Chongqing	Fruit	NA	NA
CQYC1-1-1-2	Peach	Yongchuan, Chongqing	Fruit	MN958555	MT845679
CQYC2-2-2-2	Peach	Yongchuan, Chongqing	Fruit	NA	NA
CQYC2-4-2	Peach	Yongchuan, Chongqing	Fruit	NA	NA
CQYC3-4-1-1	Peach	Yongchuan, Chongqing	Fruit	NA	NA
GDLC1-3-1	Peach	Lechang, Guangdong	Fruit	MN958564	MT845688
GDLC1-6-1	Peach	Lechang, Guangdong	Fruit	MN958565	MT845689
GDLC1-1-1	Peach	Lechang, Guangdong	Fruit	NA	NA
GDLC1-2-1	Peach	Lechang, Guangdong	Fruit	NA	NA
GDLC1-4-1	Peach	Lechang, Guangdong	Fruit	NA	NA
GDLC1-5-1	Peach	Lechang, Guangdong	Fruit	NA	NA
GXGL1-2-1	Peach	Guilin, Guangxi	Fruit	MN958566	MT845690
GXGL2-11–3	Nectarine	Guilin, Guangxi	Fruit	MN958567	MT845691
GXGL4-1-1	Peach	Guilin, Guangxi	Fruit	NA	NA
GXGL4-5-1	Peach	Guilin, Guangxi	Fruit	MN958568	MT845692
GXGL1-1-1	Peach	Guilin, Guangxi	Fruit	NA	NA
GZTR1-1-1	Peach	Guilin, Guangxi	Fruit	NA	NA
GZTR1-2-1	Peach	Guilin, Guangxi	Fruit	NA	NA
GZTR1-3-1	Peach	Guilin, Guangxi	Fruit	NA	NA
GZTR1-17–1	Peach	Tongren, Guizhou	Fruit	MN958569	MT845693
GZTR1-4-1	Peach	Tongren, Guizhou	Fruit	MN958570	MT845694
GZTR1-4-2	Peach	Tongren, Guizhou	Fruit	MN958571	MT845695
HBSJZ1-1-1	Peach	Shijiazhuang, Hebei	Fruit	NA	NA
HBSJZ1-16–1	Peach	Shijiazhuang, Hebei	Fruit	MN958573	MT845697
HBSJZ1-3-1	Peach	Shijiazhuang, Hebei	Fruit	NA	NA
HBSJZ1-2-1	Peach	Shijiazhuang, Hebei	Fruit	NA	NA
HBSJZ1-9-1	Peach	Shijiazhuang, Hebei	Fruit	MN958574	MT845698
HBSJZ1-4-1	Peach	Shijiazhuang, Hebei	Fruit	NA	NA
HBSZ1-6-1	Peach	Suizhou, Hubei	Fruit	NA	NA
HBSZ2-11–1	Peach	Suizhou, Hubei	Fruit	NA	NA
HBSZ2-3-1	Peach	Suizhou, Hubei	Fruit	NA	NA
HBSZ3-5-3	Peach	Suizhou, Hubei	Fruit	NA	NA
HBSZ5-8-3	Peach	Suizhou, Hubei	Fruit	MN958576	MT845700
HBXG1-1-1	Peach	Xiaogan, Hubei	Fruit	MN958577	MT845701
HBXG1-3-1	Peach	Xiaogan, Hubei	Fruit	NA	NA
HBXG2-19-1	Peach	Xiaogan, Hubei	Fruit	NA	NA
HBXG2-19–2	Peach	Xiaogan, Hubei	Fruit	NA	NA
HBXG2-23–1	Peach	Xiaogan, Hubei	Fruit	NA	NA
HBXG3-11–1	Peach	Xiaogan, Hubei	Fruit	MN958578	MT845702
HBXG3-6-1	Peach	Xiaogan, Hubei	Fruit	NA	NA
HBXT1-10-1	Peach	Xiantao, Hubei	Fruit	MN958579	MT845703
HBYB1-2-1	Peach	Yibing, Hubei	Fruit	MN958580	MT845704
HZAUMP1-2-1	Peach	Wuhan, Hubei	Fruit	NA	NA
HZAU13-5-1	Peach	Wuhan, Hubei	Fruit	MN958581	MT845705
HZAU3-2-2	Peach	Wuhan, Hubei	Fruit	MN958582	MT845706
HZAU3-3-1	Peach	Wuhan, Hubei	Fruit	NA	NA
HZAU5-1-1	Peach	Wuhan, Hubei	Fruit	MN958583	MT845707
HZAU5-1-2	Peach	Wuhan, Hubei	Fruit	NA	NA
HZAU6-2-1	Peach	Wuhan, Hubei	Fruit	MN958584	MT845708
HZAU8-1-2	Peach	Wuhan, Hubei	Fruit	NA	NA
HNJZ1-1-1-1	Peach	Jiaozuo, Henan	Fruit	MN958585	MT845709
HNJZ1-3-1	Peach	Jiaozuo, Henan	Fruit	NA	NA
HNXZ1-3-1	Peach	Xinzheng, Henan	Fruit	MN958586	MT845710
HNXZ2-1-1	Peach	Xinzheng, Henan	Fruit	NA	NA
HNZMD1-3-1	Peach	Zhumadian, Henan	Fruit	MN958587	MT845711
JSNJ1-3-1	Peach	Nanjin, Jiangsu	Fruit	MN958588	MT845712
JSSQ2-4-1	Peach	Suqian, Jiangsu	Fruit	MN958589	MT845713
JSSQ3-2-1	Peach	Suqian, Jiangsu	Fruit	NA	NA
JSSQ4-1-1	Nectarine	Suqian, Jiangsu	Fruit	NA	NA
JSSQ5-1-1	Peach	Suqian, Jiangsu	Fruit	MN958590	MT845714
JSSQ6-1-1	Nectarine	Suqian, Jiangsu	Fruit	MN958591	MT845715
JSSQ7-3-1	Peach	Suqian, Jiangsu	Fruit	NA	NA
SCCD1-2-3	Peach	Chengdu, Sichuan	Fruit	MN958592	MT845716
SCCD1-4-3	Peach	Chengdu, Sichuan	Fruit	NA	NA
SCCD3-1-2	Nectarine	Chengdu, Sichuan	Fruit	MN958593	MT845717
SCCD4-1-2	Peach	Chengdu, Sichuan	Fruit	NA	NA
SCCD5-1-2	Peach	Chengdu, Sichuan	Fruit	NA	NA
SCCD6-2-2	Peach	Chengdu, Sichuan	Fruit	NA	NA
SCCD8-1-2	Peach	Chengdu, Sichuan	Fruit	MN958594	MT845718
SCJY1-1-1	Peach	Jianyang, Sichuan	Fruit	NA	NA
SCJY1-7-2	Peach	Jianyang, Sichuan	Fruit	MN958595	MT845719
SCJY2-1-1	Peach	Jianyang, Sichuan	Fruit	MN958596	MT845720
SDLX1-10-2	Peach	Laixi, Shandong	Fruit	NA	NA
SDLX1-1-3	Nectarine	Laixi, Shandong	Fruit	MN958597	MT845721
SDLX1-7-2	Peach	Linyi, Shandong	Fruit	NA	NA
SDLY1-1-1	Peach	Linyi, Shandong	Fruit	NA	NA
SDLY1-4-1	Peach	Linyi, Shandong	Fruit	MN958598	MT845722
SDLY1-6-1	Peach	Linyi, Shandong	Fruit	NA	NA
SDQD1-1-4	Peach	Qingdao, Shandong	Fruit	MN958599	MT845723
SDQD1-5-2	Peach	Qingdao, Shandong	Fruit	NA	NA
SDQD1-6-3	Peach	Qingdao, Shandong	Fruit	NA	NA
SDTA1-1-2	Peach	Taian, Shandong	Fruit	NA	NA
SDTA2-10-1	Peach	Taian, Shandong	Fruit	MN958600	MT845724
SDTA2-5-3	Peach	Taian, Shandong	Fruit	NA	NA
SDWF1-1-2	Peach	Weifang, Shandong	Fruit	MN958601	MT845725
SDWF1-5-4	Peach	Weifang, Shandong	Fruit	NA	NA
SDWF2-4-1	Peach	Weifang, Shandong	Fruit	NA	NA
SXXA1-1-1	Nectarine	Xi’an, Shaanxi	Fruit	NA	NA
SXXA1-3-1	Nectarine	Xi’an, Shaanxi	Fruit	NA	NA
SXXA1-10-1	Nectarine	Xi’an, Shaanxi	Fruit	MN958602	MT845726
SXXA1-2-1	Nectarine	Xi’an, Shaanxi	Fruit	NA	NA
SXXA1-8-1	Nectarine	Xi’an, Shaanxi	Fruit	MN958603	MT845727
SXXA2-1-1	Peach	Xi’an, Shaanxi	Fruit	MN958604	NA
YNKM1-2-1	Peach	Kunming, Yunnan	Fruit	NA	NA
YNKM1-2-2	Peach	Kunming, Yunnan	Fruit	MN958605	MT845728
YNKM1-2-3	Peach	Kunming, Yunnan	Fruit	NA	NA
YNKM1-4-1	Peach	Kunming, Yunnan	Fruit	MN958606	MT845729
YNKM1-5-1	Peach	Kunming, Yunnan	Fruit	MN958607	MT845730
ZJHZ10-1-1	Peach	Hangzhou, Zhejiang	Fruit	MN958608	MT845731
ZJHZ1-1-1	Peach	Hangzhou, Zhejiang	Fruit	MN958609	MT772296
ZJHZ11-4	Peach	Hangzhou, Zhejiang	Fruit	MN958610	MT845732
ZJHZ1-2-2	Peach	Hangzhou, Zhejiang	Fruit	MN958611	MT845733
ZJHZ12-2-1	Peach	Hangzhou, Zhejiang	Fruit	NA	NA
ZJHZ13-2-1	Nectarine	Hangzhou, Zhejiang	Fruit	MN958612	MT845734
ZJHZ1-4-1	Peach	Hangzhou, Zhejiang	Fruit	MN958615	MT845737
ZJHZ14-1-1	Peach	Hangzhou, Zhejiang	Fruit	MN958613	MT845735
ZJHZ14-1-2	Peach	Hangzhou, Zhejiang	Fruit	NA	NA
ZJHZ15-2-1	Nectarine	Hangzhou, Zhejiang	Fruit	MN958614	MT845736
ZJHZ19-3-1	Peach	Hangzhou, Zhejiang	Fruit	MN958616	MT845738
ZJHZ2-2-1	Peach	Hangzhou, Zhejiang	Fruit	MN958617	MT845739
ZJHZ2-2-1	Peach	Hangzhou, Zhejiang	Fruit	NA	NA
ZJHZ2-2-2	Peach	Hangzhou, Zhejiang	Fruit	NA	NA
ZJHZ2-3-1	Peach	Hangzhou, Zhejiang	Fruit	MN958618	MT845740
ZJHZ2-4-3	Peach	Hangzhou, Zhejiang	Fruit	NA	NA
ZJHZ2-5-1	Peach	Hangzhou, Zhejiang	Fruit	MN958619	MT845741
ZJHZ3-1-1	Peach	Hangzhou, Zhejiang	Fruit	MN958620	MT845742
ZJHZ4-2-1	Peach	Hangzhou, Zhejiang	Fruit	NA	NA
ZJHZ4-2-2	Peach	Hangzhou, Zhejiang	Fruit	MN958621	MT845743
ZJHZ5-3-1	Peach	Hangzhou, Zhejiang	Fruit	MN958622	MT845744
ZJHZ7-2-1	Peach	Hangzhou, Zhejiang	Fruit	NA	NA
ZJHZ7-2-3	Peach	Hangzhou, Zhejiang	Fruit	MN958623	MT845745
ZJHZ7-2-5	Peach	Hangzhou, Zhejiang	Fruit	NA	NA
ZJHZ8-2-3	Peach	Hangzhou, Zhejiang	Fruit	MN958624	MT845746
ZJHZ8-3-1	Peach	Hangzhou, Zhejiang	Fruit	NA	NA
ZJHZ9-2-1	Nectarine	Hangzhou, Zhejiang	Fruit	MN958625	MT845747
HZAUTY4-1-2	Peach	Wuhan, Hubei	Leaf	MT995091	MT995106
HZAUTY3-1-1	Peach	Wuhan, Hubei	Leaf	MT995092	MT995107
HZAUTY3-1-2	Peach	Wuhan, Hubei	Leaf	MT995093	MT995108
HZAUTY2-1-1	Peach	Wuhan, Hubei	Leaf	MT995094	MT995109
HZAUMPTY7-1-1	Peach	Wuhan, Hubei	Leaf	MT995095	MT995110
HZAUMPTZ6-1-1	Peach	Wuhan, Hubei	Twig	MT995096	MT995111
HZAUMPTZ6-1-3	Peach	Wuhan, Hubei	Twig	MT995097	MT995112
HZAUMPTZ7-1-1	Peach	Wuhan, Hubei	Twig	MT995098	MT995113
HZAUMPTZ8-1-2	Peach	Wuhan, Hubei	Twig	MT995099	MT995114
HZAUTZ3-1-1	Peach	Wuhan, Hubei	Twig	MT995100	MT995115
HZAUTG4-1-2	Peach	Wuhan, Hubei	Fruit	MT995101	MT995116
HZAUTG3-1-1	Peach	Wuhan, Hubei	Fruit	MT995102	MT995117
HZAUTG2-1-2	Peach	Wuhan, Hubei	Fruit	MT995103	MT995118
HZAUTG2-1-1	Peach	Wuhan, Hubei	Fruit	MT995104	MT995119
HZAUTG1-1-1	Peach	Wuhan, Hubei	Fruit	MT995105	MT995120
HZAUMZ4-1-1	Mume	Wuhan, Hubei	Twig	NA	NA
HZAUMZ3-1-2	Mume	Wuhan, Hubei	Twig	MN958630	MT845752
HZAUMZ3-1-1	Mume	Wuhan, Hubei	Twig	MN958631	MT845753
HZAUMZ1-1-2	Mume	Wuhan, Hubei	Twig	MN958632	MT845754
HZAUMZ1-1-1	Mume	Wuhan, Hubei	Twig	NA	NA
HZAUMY6-1-1	Mume	Wuhan, Hubei	Leaf	MN958633	MT845755
HZAUMY4-1-1	Mume	Wuhan, Hubei	Leaf	NA	NA
HZAUMY1-1-2	Mume	Wuhan, Hubei	Leaf	NA	NA
HZAUMY3-1-1	Mume	Wuhan, Hubei	Leaf	MN958634	MT845756
HZAUMY1-1-1	Mume	Wuhan, Hubei	Leaf	MN958635	MT845757
HZAUMG6-1-1	Mume	Wuhan, Hubei	Fruit	MN958636	MT845758
HZAUMG4-1-1	Mume	Wuhan, Hubei	Fruit	NA	NA
HZAUMG3-1-1	Mume	Wuhan, Hubei	Fruit	MN958637	MT845759
HZAUMG1-1-2	Mume	Wuhan, Hubei	Fruit	NA	NA
HZAUMG1-1-1	Mume	Wuhan, Hubei	Fruit	MN958638	MT845760
HZAUXG5-1-2	Apricot	Wuhan, Hubei	Fruit	MN958639	MT845761
HZAUXG5-1-1	Apricot	Wuhan, Hubei	Fruit	NA	NA
HZAUXG3-1-2	Apricot	Wuhan, Hubei	Fruit	MN958640	MT845762
HZAUXG2-1-1	Apricot	Wuhan, Hubei	Fruit	NA	NA
HZAUXG1-1-1	Apricot	Wuhan, Hubei	Fruit	MN958641	MT845763
HZAUXZ5-1-2	Apricot	Wuhan, Hubei	Twig	MN958642	MT845764
HZAUXZ4-1-2	Apricot	Wuhan, Hubei	Twig	NA	NA
HZAUXZ4-1-1	Apricot	Wuhan, Hubei	Twig	MN958643	MT845765
HZAUXZ1-1-1	Apricot	Wuhan, Hubei	Twig	MN958644	MT845766
HZAUXY5-1-2	Apricot	Wuhan, Hubei	Leaf	MN958645	MT845767
HZAUXY5-1-1	Apricot	Wuhan, Hubei	Leaf	NA	NA
HZAUXY3-1-2	Apricot	Wuhan, Hubei	Leaf	MN958646	MT845768
HZAUXY2-1-1	Apricot	Wuhan, Hubei	Leaf	NA	NA
HZAUXY1-1-1	Apricot	Wuhan, Hubei	Leaf	MN958647	MT845769
HZAUXZ2-1-1	Apricot	Wuhan, Hubei	Leaf	NA	NA

a NA, Data not uploaded, only select the sequences of some representative isolates were uploaded to NCBI. rDNA-ITS, internal transcribed spacer regions; *LSU*, large subunit ribosomal RNA gene.

### DNA extraction

Suspension of *V. carpophila* spores was transferred into 50 ml of malt extract broth (MEB, 30 g of malt extract and 5 g of peptone per liter of water) in an Erlenmeyer flask, and incubated at 21°C, 160 rpm for 10 days to obtain a sufficient amount of mycelium for DNA extraction. Approximately 100 mg of mycelium of each isolate was collected from the MEB broth, and put into a pilot freeze dryer (25 L Genesis SQ Super ES-55) to dehydrate for 48 h. Dehydrated hyphae were placed into Eppendorf tubes containing 0.5 g steel balls (5 mm in diameter) and frozen with liquid nitrogen for 2 min. Mycelium was homogenized by shaking the tubes for 5 min at 30 shakes/s in a Mixer Mill Type LT2020 (Beijing Dinghaoyuan Gmbh, China). After homogenization, DNA was immediately extracted by the modified SDS method (Turan et al., 2015), and genomic DNA was diluted with 70 μl of sterile distilled water. DNA was quantified with a NanoDrop spectrophotometer (Thermo Fisher Scientific) and stored at −20°C.

### Identification of isolates

Isolates of *V. carpophila* were identified based on morphological and molecular characterization. Briefly, fragments of nuclear ribosomal internal transcribed spacer regions (rDNA-ITS; White et al., 1990) and large subunit ribosomal RNA gene (*LSU*; Vilgalys and Hester, 1990) were amplified from 186 isolates by polymerase chain reaction (PCR), and then used for sequencing. The PCR reaction was performed following the conditions of 95°C for 3 min, followed by 35 cycles at 95°C for 30 s, annealing at 55°C for 50 s, 72°C for 2 min, and 72°C for 5 min. The Sanger sequencing of PCR products was performed on ABI 3730xl DNA Sequencer at Tianyihuiyuan Biotechnology Co., Ltd. (Wuhan, China). Phylogenetic trees of *Venturia* species were constructed based on the combined multi-locus dataset (*LSU* + ITS). The majority rule consensus tree from maximum parsimony (MP) analysis was used to show the phylogenetic relationships of *Venturia* species and their close species. The tree was rooted with *Sympoventuria capensis* (CBS 120136). The Bayesian posterior probabilities values (BIPP) ≥0.70, maximum likelihood and maximum parsimony bootstrap values (MLBS and MPBS) ≥ 50% were shown at the branch nodes (BIPP/MLBS/MPBS).

### Analysis of genetic diversity and population structure of *Venturia carpophila*

The genetic diversity analysis was carried out according to the previous description with minor modifications (Chen et al., 2018). Thirty-two microsatellite primers (Supplementary Table S1) were developed from a draft genome of *V. carpophila* which has been released, after primary screening, 31 microsatellite primers were used to conduct the genetic diversity analysis in this study (Chen et al., 2018). SSR markers were obtained *via* PCR reactions with fluorescently labeled primers. Primers were manufactured at Tianyihuiyuan Biotechnology Co., Ltd. (Wuhan, China). When primers were synthesized, the universal M13 sequence (5′-TGT AAA ACG ACG GCC AGT-3′) was added to the 5′ end of each forward primer. Simultaneously, M13 was labeled by FAM fluorescent dyes at the 3′ end. The labeled M13 was added to the PCR reaction to detect PCR product by complementing the unlabeled M13 added at 5′ end of primer (Chen et al., 2018). The PCR reaction was in a volume of 15 μl, including 4.5 μl ddH_2_O, 7.5 μl Mastermix (no dye), 0.4 μl of M13-tagged forward primer (10 μM) and 0.8 μl reverse primer (10 μM), 0.8 μl FAM-labeled M13 primer (10 μM), and 1 μl DNA (50~200 ng/μl). PCR conditions were an initial denaturation at 95°C for 5 min followed by 10 cycles of denaturation at 95°C for 30 s, annealing at 62°C for 30 s (with a −1°C Ramp/cycle), and extension at 72°C for 30 s, a further 25 cycles of denaturation at 95°C for 30 s, annealing at 52°C for 30 s, extension 72°C for 30 s, and ending with a final extension of 72°C for 2 min (iCycler; Bio-Rad Laboratories Inc., Hercules, CA). The fluorescent PCR products were loaded to an ABI 3730 × I Genetic Analyzer Capillary to generate chromatographic trace files. Negative control (ddH_2_O) was included in the analysis to eliminate potential contamination. Resulted amplicon peaks were scored by base pair size for each marker using GeneMarker V2.2.0 to generate a microsatellite allele table for subsequent analysis. The PCR-generated bands were scored as “1” (for presence) and “0” (absence) in a binary matrix for further analysis. Because the *V. carpophila* is a haploid organism, only a single band was observed at each locus for individual isolate.

### Data analysis

The scorable bands for all isolates were determined and analyzed as dominant markers with genetics based on a haploid organism (Bock et al., 2005; Chen et al., 2014). The number of markers observed for the 186 isolates with each primer set was counted, and the percentage of polymorphic loci was calculated. The numbers of alleles (*Na*), and effective alleles (*Ne*) were calculated using Popgene 32 based on SSR markers identified among isolates of *V. carpophila* (Yeh et al., 1999).

The genetic structure of the populations was further explored by using analysis of molecular variance (AMOVA) in GenALEX 6.5, which was conducted at two scales: across geographic populations, across host populations (Peakall and Smouse, 2006). The population structure of *V. carpophila* was analyzed in five ways. (1) A principal coordinate analysis (PCoA) was performed to visualize patterns of variation between populations at the host and geographic region levels based on Nei’s measure of genetic distance, in GenALEX 6.5 (Peakall and Smouse, 2006). (2) STRUCTURE 2.2 was used to detect the most likely number of populations (K) among the *V. carpophila* isolates based on allele frequencies per locus using a Bayesian approach (Falush et al., 2007). To estimate the number of clusters, an admixture model with correlated allele frequencies was independently run 10 times, with 10,000 iterations followed by 120,000 Markov chain Monte Carlo interactions (Bock et al., 2014). Evanno’s ΔK method was used to identify the appropriate number of clusters (Evanno et al., 2005), which was computed using Structure Harvester (Earl and vonHoldt, 2012). (3) Discriminant analysis of principal components (DAPC) was performed in adegenet of R software (Jombart et al., 2010), thus interrelationships among *V. carpophila* populations from different geographic regions or different hosts could be visually explored. (4) The minimum spanning network (MSN) was created in the R package *poppr* (Bruvo et al., 2004) by using default parameters to identify the distribution of population substructure at different scales. (5) To compare populations, the UPGMA analysis of population structure based on Nei’s genetic distance was calculated using MEGA7, and annotation and management of phylogenetic trees were conducted using Evolview V2 (He et al., 2016).

Significant differences of growth rate and spore production values from different host populations were evaluated by one-way analysis of variance with the least significant difference test in SPSS software (release 19.0; SPSS).

## Results

### Identification and genotyping of *Venturia carpophila*

**Figure 3:**
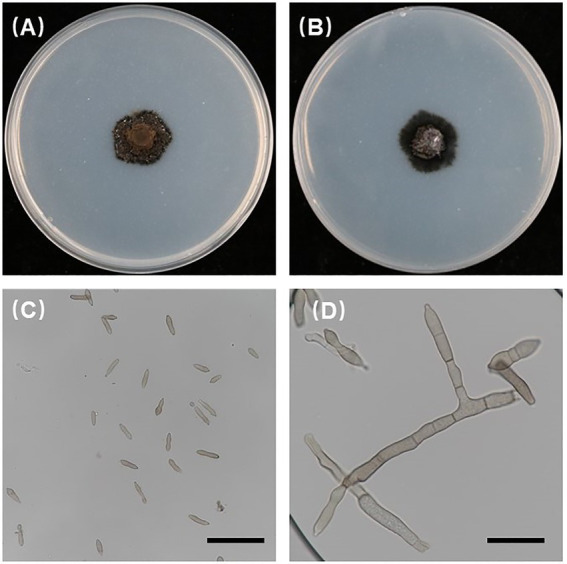
Morphological characteristics of *Venturia carpophila.* **(A,B)** Front and back view of 42-day-old PDA culture (petri dish is 90 mm); **(C,D)** conidia and conidiophores produced on PDA. Scale bars: **C** = 40 μm; **D** = 20 μm.

In the current study, all 186 isolates used were investigated for morphological characteristics. The 186 isolates were incubated at 21°C for 6 weeks in darkness on PDA, and the *V. carpophila* colonies on PDA were black with circular morphology and floccose texture (Figures 3A,B). The conidia were cylindrical to fusiform, 0 to 1 septate, yellowish and (11.44 to 23.16) 15.87 × 4.15 (2.56 to 6.48) μm (*n* = 30) in size, L/W = 3.8 (Figures 3C,D), conidiophore is fusiform (Figure 3D), which were similar with the characteristics of *V. carpophila* described previously (Chen et al., 2017; Dar et al., 2019; Shen et al., 2020).

**Figure 4:**
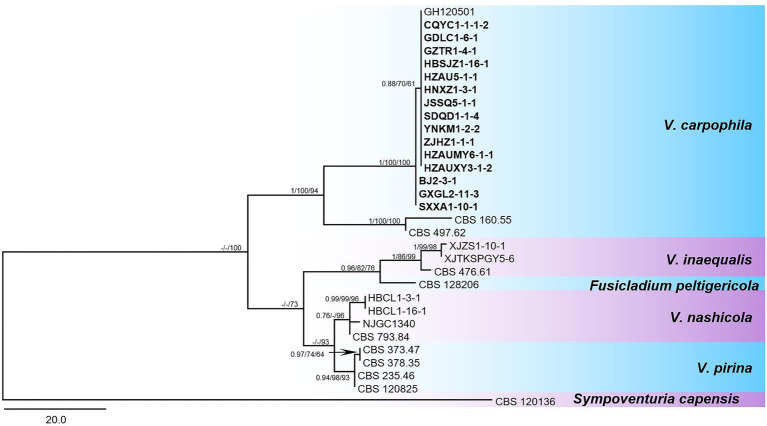
Phylogenetic tree in the *Venturia* species inferred from combined alignment with a two-locus dataset (*LSU +* ITS). The maximum parsimony (MP) analysis was shown for the phylogenetic relationships of species among *Venturia* spp. and closely related species. The tree was rooted to *Sympoventuria capensis* (CBS 120136). The Bayesian posterior probabilities values (BIPP) > 0.70, maximum likelihood and maximum parsimony bootstrap values (MLBS and MPBS) > 50% were shown at the branch nodes (BIPP/MLBS/MPBS). Scale bar estimated substitutions per site with 20. The isolates used in this study were displayed in bold fonts.

The phylogenetic analysis based on sequences of fragments from two loci (*LSU* + ITS) showed that the tested isolates grouped with representative *V. carpophila* strains GH120501 (Korea), CBS160.55 (United States), and CBS497.62 (Switzerland) in the same clade (Figure 4). New sequences obtained in this study were deposited in GenBank (Table 1, ITS: MN958548 to MN958647; LSU: MT845672 to MT845769).

These 186 isolates were genotyped based on the ITS region which was amplified using the ITS4 and ITS5 primers, and four haplotypes were found for the ITS region with one to two nucleotide changes (Supplementary Figure S1). Overall, most of the isolates fell into ITS haplotype group I (MN958568, MN958609), group II (MN958640) with 82.80, and 15.59%, respectively. Only 1.08, and 0.54% of the tested isolates were in haplotype group III (MN958584), and group IV (MN958592).

### Screening of the SSR markers

To better understand the genetic diversity and population structure of Chinese *V. carpophila* populations, 31 SSR primer sets were chosen to analyze 186 selected single spore isolates. One peak per isolate, consistent with the haploid nature of the *V. carpophila*, was observed for all the loci. Among the tested 31 SSR primer sets, two primer sets (Vc015 and Vc021) showed monomorphic profiles with the only peaks at 222 bp (Vc015) and 301 bp (Vc021). The remaining 29 primer sets showed polymorphic profiles, the number of alleles at each microsatellite locus ranged from two to 21, and a total of 135 alleles were identified, with a mean number of 4.66 alleles per locus. Vc110 was the most variable primer set with 21 alleles detected and polymorphism information content (PIC) value was 0.77, whereas Vc106 had only 2 alleles and PIC value was 0.03 (Supplementary Table S1). The genotype accumulation curve showed that 29 polymorphic microsatellites were adequate to describe the genetic variability of *V. carpophila* (Supplementary Figure S2).

### Genetic diversity of *Venturia carpophila* isolates

Altogether a total of 135 polymorphic loci (*NPL*) were detected in 186 isolates. Among them, 105 *NPL* were detected in 14 geographic populations and 102 *NPL* were detected in three host populations (Supplementary Tables S2, S3). In geographic populations, Hubei (HuB) had the largest number of *NPL* (77), and Hebei (HeB) had the least (22). In host populations, apricot had the largest number of *NPL* (77), and mume had the least number (44). Different levels of genetic diversity (*H*) were observed among 14 geographic populations and 3 host populations, ranging from 0.07 for HeB population to 0.18 for HuB population; a low genetic diversity of mume isolates (0.11) was observed, while higher genetic diversities were detected for peach isolates (0.14) and apricot isolates (0.17). Geographic populations showed a low percentage of polymorphic loci (*PPL*) in HeB population (15.30% of markers) but a higher *PPL* in Zhejiang (ZJ) population (50.37%) and HuB population (57.04%). Host populations showed a low *PPL* in mume population (32.59% of markers), and higher *PPL* in peach population (42.96%) and apricot population (57.05%). The number of observed alleles (*Na*) of the apricot population and HuB population were up to 1.57. The effective allele number (*Ne*) and Shannon index (*I*) of the HuB population were the highest, i.e., 1.31 and 0.27, respectively. Interestingly, the apricot population had only 15 isolates, but the number of polymorphic loci (*NPL*) was 77, as high as the HuB population (37 isolates; Supplementary Tables S2, S3). These results indicated that genetic diversity varied among different populations, and the HuB population showed the highest genetic diversity among different geographic populations, while the apricot population had the highest genetic diversity among different host populations.

### Distribution of genetic variation

Among different geographic populations, the lowest genetic similarity coefficient was 0.8819 for Guizhou versus Guangdong (GZ vs. GD), and the highest was 0.9882 for Zhejiang versus Hubei (ZJ vs. HuB). Among different host populations, the lowest genetic similarity coefficient was 0.8616 for apricot vs. mume (A vs. M), and the highest was 0.8841 for peach vs. mume (P vs. M, Supplementary Tables S4, S5). These results indicated that populations shared a moderate to substantial identity. At the same time, Nei’s genetic distance indicated that geographic populations varied in their degree of relatedness from 0.0118 (HuB vs. ZJ), which was separated by a relatively short genetic distance, to 0.1257 (GD vs. GZ), which was separated by greater genetic distance (Supplementary Table S4). Different host populations varied in their degree of relatedness from 0.1231 (P vs. M) to 0.149 (M vs. A; Supplementary Table S5).

### Genetic differentiation of *Venturia carpophila*

AMOVA analysis of 14 geographic populations indicated that 13% of the observed genetic diversity was partitioned among the populations and 87% was within individual populations (PhiPT = 0.125; *p* = 0.001). For three host populations, 40% of the observed genetic diversity was partitioned among the populations and 60% was within individual populations (PhiPT = 0.403; *p* = 0.001). A moderate genetic differentiation was observed across host populations, however, limited genetic differentiation was observed across geographic populations (Supplementary Tables S5, S6). The pairwise indices of differentiation quantified the genetic variation between geographic and host populations (Supplementary Tables S6, S7). Significant pairwise geographic population PhiPT values ranged from 0.00 to 0.509, and the highest differentiation was found between the HeB and Yunnan (YN) populations (Supplementary Table S8). Significant pairwise host population PhiPT values ranged from 0.367 to 0.426, and the highest differentiation was found between the mume and apricot populations (Supplementary Table S9).

### Population structure

**Figure 5:**
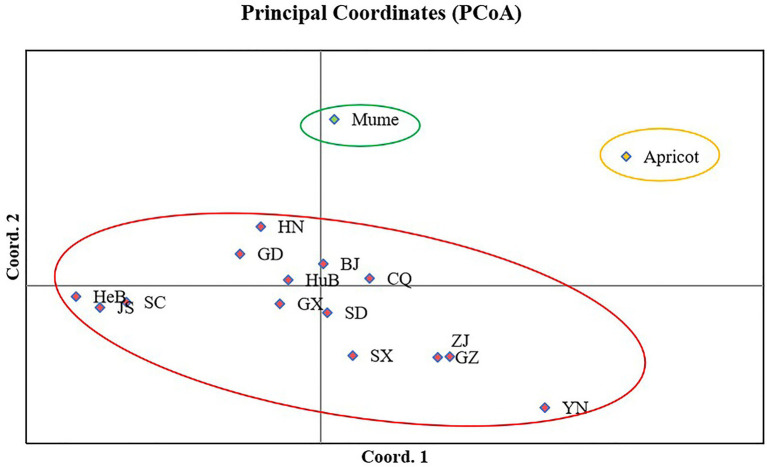
Principal coordinate analysis of 16 populations of *Venturia carpophila* in China based on Nei’s genetic distance using GenALEX. The red circle represents the isolates from peach, the green circle represents the isolates from mume and yellow circle represents the isolates from apricot. BJ, Beijing; CQ, Chongqing; GD, Guangdong; GX, Guangxi; GZ, Guizhou; HeB, Hebei; HuB, Hubei; HN, Henan; JS, Jiangsu; SC, Sichuan; SD, Shandong; SX, Shanxi; YN, Yunnan; ZJ, Zhejiang.

Principle coordinate analysis (PCoA) is a multidimensional scaling analysis to visualize the clustering of isolates due to genetic similarities among tested isolates. The analysis was performed in GenAlEx V6.5 based on Nei’s genetic distance. PCoA revealed three groups, one was specific to apricot isolates, the other was to mume isolates, and the third one was to peach isolates. Axes 1 and 2 of the PCoA accounted for 29.75% and 25.12% of the total genetic variation. Isolates from mume were relatively closer to peach isolates (Figure 5).

**Figure 6:**
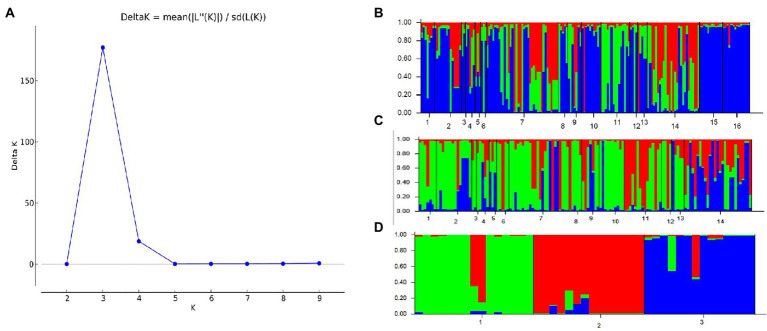
Estimation of population structure. **(A)** Optimum number of subpopulations was determined by using LnP(D) derived ∆K. The maximum value of delta K was found to be at K = 3, suggesting division of the entire population into 3 subpopulations. **(B)** Assignment plots of *Venturia carpophila* from 14 peach producing provinces and 3 hosts with K = 3 performed in the program STRUCTURE. Black lines separate isolates from different locations. 1 = Beijing (BJ), 2 = Chongqing (CQ), 3 = Guangdong (GD), 4 = Guangxi (GX), 5 = Guizhou (GZ), 6 = Hebei (HeB), 7 = Hubei (HuB), 8 = Henan (HN), 9 = Jiangsu (JS), 10 = Sichuan (SC), 11 = Shandong (SD), 12 = Shanxi (SX), 13 = Yunnan (YN), 14 = Zhejiang (ZJ), 15 = Mume, 16 = Apricot. **(C)** Assignment plots of *V. carpophila* from 14 peach producing provinces with K = 3 performed in the program STRUCTURE. 1 = Beijing, 2 = Chongqing, 3 = Guangdong, 4 = Guangxi, 5 = Guizhou, 6 = Hebei, 7 = Hubei, 8 = Henan, 9 = Jiangsu, 10 = Sichuan, 11 = Shandong, 12 = Shanxi, 13 = Yunnan, 14 = Zhejiang. **(D)** Assignment plots of *V. carpophila* from 3 hosts with K = 3 performed in the program STRUCTURE, 1 = Peach, 2 = Mume, 3 = Apricot.

**Figure 7:**
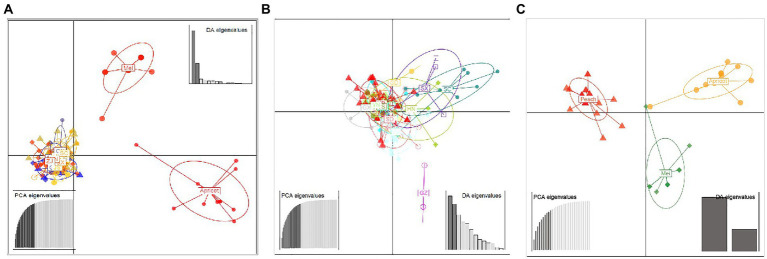
Discriminant analysis of principal components (DAPC) of *Venturia carpophila* populations including different geographic and host populations **(A)**. Discriminant analysis of principal components of 14 geographic *V. carpophila* populations on peach **(B)**. Discriminant analysis of principal components of 3 host populations (Peach, mume and apricot) *V. carpophila* **(C)**. Eigenvalues signifying the variance explained by principal component analysis (PCA) and discriminant analysis (DA) indicated that the first two principal components adequately explain the genetic structure of the populations. Points represent individual isolates and ellipses represent individual populations. There is no representation of clonal individuals in this graphic.

**Figure 8:**
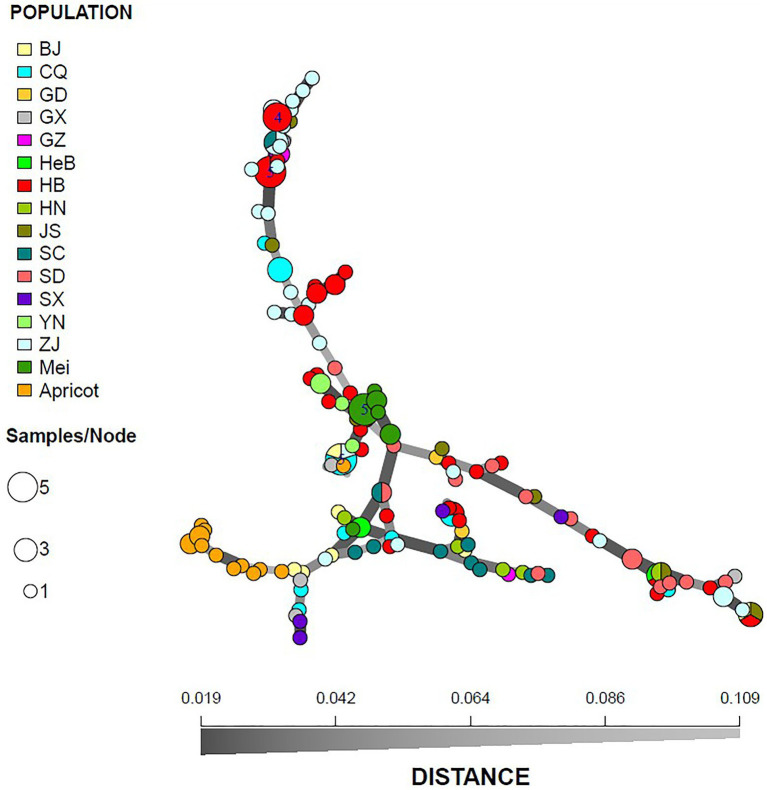
Minimum spanning network of 186 *Venturia carpophila* based on genetic distance from 14 geographic and 3 host populations. Node colors represent population membership and are proportional to the pie size. There is no representation of clonal individuals in this graphic.

**Figure 9:**
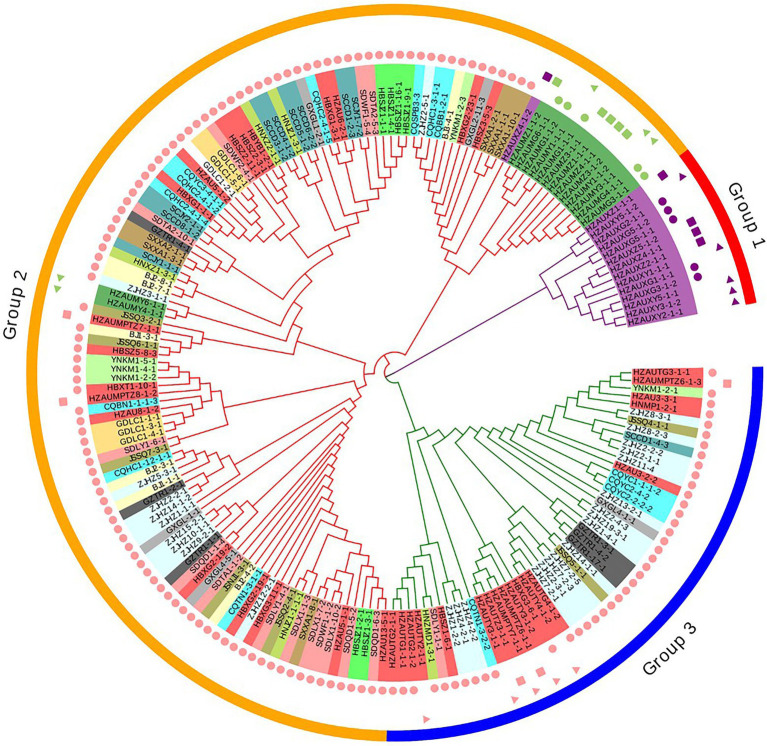
Relationships among 186 *Venturia carpophila* isolates from 14 provinces and 3 hosts. The dendrogram was constructed using the unweighted pair group method with arithmetic mean based on Nei’s genetic distance. Shape labels, circle: isolates from fruits, rectangle: isolates from twigs and triangle: isolates from leaves. Color labels, pink: isolates from peach, green: isolates from mume and purple: isolates from apricot. Phylogenetic tree was managed and annotated by using Evolview V2.

A similar clustering pattern was obtained by structure analyses. Structure analysis of all of the populations revealed that the highest likelihood values and the mode of the distribution of the ΔK index were all observed for K = 3 (Figure 6A), i.e., 186 isolates *V. carpophila* from China were divided into three subgroups (K = 3; Figure 6B). Structure analysis of 14 geographic populations revealed that the highest likelihood values and the mode of the distribution of the ΔK index were all observed for K = 3 (Same as Figure 6A, data not shown), significantly higher than other clusters. From 14 geographic populations, isolates were divided into three subgroups (K = 3; Figure 6C) which were not correlated with geographic regions. Structure analysis of 3 host populations revealed that the highest likelihood values and the mode of the distribution of the ΔK index were all observed for K = 3 (data not shown), significantly higher than other clusters. From 3 host populations, isolates were divided into three subgroups (K = 3; Figure 6D) which were correlated with hosts, i.e., isolates from the same host clustered in a group. Similarly, the DAPC analysis displayed the interrelationship of the 186 Chinese *V. carpophila* haplotypes. The model-based DAPC analyses suggested the existence of at least three clusters in *V. carpophila* populations (Figure 7A). One hundred and fifty-six isolates collected from 14 provinces on peach were overlapped (Figure 7B), while 45 isolates from different 3 hosts at the same collection geographic region showed that there are three clusters corresponding to their specific hosts (Figure 7C). Minimum spanning network (MSN) is a great way to visualize relationships among individual isolates. It is a more powerful visualization tool than trees. MSN analyses further confirmed the subdivision of populations based on hosts, where the apricot population was obviously separated from other populations, and mume isolates were relatively closer to peach isolates (Figure 8). The dendrogram was inferred using the UPGMA method based on a pairwise comparison genetic distance between populations, demonstrated three major clusters of sampling populations: one cluster included all isolates from apricot, the second cluster included all isolates from mume and a few isolates from peach, and the third cluster were all peach isolates (Figure 9). Altogether, the population structure of Chinese *V. carpophila* was analyzed in five ways, i.e., PCoA, STRUCTURE, UPGMA, MSN, and DAPC, and congruence among PCoA, DAPC, UPGMA, MSN, and STRUCTURE clusters was observed. The *V. carpophila* populations in China were clustered based on specific hosts instead of the different geographic regions.

## Discussion

In this study, all of the isolates from scab samples of peach, mume, and apricot were confirmed as *V. carpophila* based on morphological and molecular characterizations. In order to better understand Chinese *V. carpophila* populations, SSR markers developed previously were used to analyze *V. carpophila* populations in China. Altogether 29 SSR markers could successfully differentiate a vast majority of isolates and sufficiently describe the genetic diversity of *V. carpophila* populations (Supplementary Figure S2). The relationship among different geographic populations and different host populations provides a new perspective for understanding the spread and inheritance of *V. carpophila*.

Chen et al. released the genomic data of *V. carpophila*, and designed SSR primers based on the genomic data (Chen et al., 2018). After screening, 32 pairs of SSR primers with polymorphisms were finally obtained. Genetic diversity analysis of 39 strains isolated from peach or nectarine using the above primers showed that the number of alleles identified ranged from 2 to 9, and the polymorphic information contents were from 0.097 to 0.792, indicating the substantial diversity of *V. carpophila* (Chen et al., 2018). Similarly, it was found that *V. carpophila* isolates in China were genetically diverse. The gene diversity of *V. carpophila* observed in this study is 0.07 to 0.18, which is similar to the results obtained in close fungal pathogen *Fusicladium effusum* (Nei’s measure of gene diversity = 0.083 to 0.160; Bock et al., 2014). The genetic diversity among *V. carpophila* isolates in China is higher than that observed in the United States (Chen et al., 2018), probably because our samples were from more geographic locations and different hosts, and also in our study, more isolates and primers were used.

Genetic diversity in host populations and geographic populations showed different levels. Genetic diversity levels among geographic populations directly correspond to the number of isolates and sampling range. For instance, the genetic diversity of the HuB population was the highest at 0.18, and the HuB population had the most isolates (37) and the widest sampling range (5 orchards that were more than 500 km apart). HeB population had the least number of isolates, only one sampling site, and showed the lowest genetic diversity at only 0.10. On the other hand, this study detected the highest genetic diversity within a small sample of 15 apricot isolates from the same orchard. Among three host populations collected from HZAU, the genetic diversity of the apricot population was higher than peach and mume populations. The sexual stage of *V. carpophila* was first reported in Australia on apricot trees in 1961. It was showed that *V. carpophila* had the ability of gene recombination (Fisher, 1961). According to our observation, the apricot leaf scab is the most common one among the three host scabs, and lesions on leaves are rare on peaches and plums. The infestation time is very long, and apricot leaf scab can be still observed until the leaf fall in autumn, which provides the basis for *V. carpophila* to produce sexual stage and genetic recombination. It might be the reason why the highest genetic diversity was observed within the apricot population.

In this study, AMOVA analysis of 14 geographic populations indicated that 13% of the observed genetic diversity was partitioned among geographic populations and 87% was within geographic populations. In addition, AMOVA analysis of three host populations indicated that 40% of the observed genetic diversity was partitioned among host populations and 60% was within host populations. This is similar to the results observed in a recent study (Bock et al., 2020). In four populations with more than 10 isolates, the AMOVA analysis indicated that most variance could be found within orchards for both original (83.10%) and clone corrected (87.98%) data sets (Bock et al., 2020). However, they did not conduct a population genetic study on isolates from different hosts. The genetic diversity was analyzed for *V. carpophila* isolates collected from peach and nectarine in the United States, although the sample size was smaller (*n* = 39 and 81), both studies revealed that the genetic diversity of *V. carpophila* isolates does not correspond to locations or host cultivars (Chen et al., 2018; Bock et al., 2020). Gene diversity is indicative that there is a transfer of genetic material among the populations infecting peach and nectarine. This may not be surprising as nectarine is considered a smooth-skinned peach (*P. persica*).

Population structure analysis revealed moderate host population differentiation (40%), which was identified when three host populations from HZAU were used. However, structure analysis of 14 geographic regions’ population revealed that *V. carpophila* were divided into three subgroups, and no significant differentiation was identified among geographic populations (13%). Analysis of the genetic structure of 81 peach isolates of *V. carpophila* from the eastern United States showed that *V. carpophila* population clustered in three distinct groups (Bock et al., 2020). In this study, the genetic structure analysis of *V. carpophila* isolates from peach (14 provinces) of China obtained similar results. Overall, these results suggested that there was a significant interflow of *V. carpophila* isolates among different geographic regions but not among different hosts, e.g., peach, mume, and apricot. Similarly, based on the DAPC analysis, isolates collected from 14 provinces did not cluster according to geographic regions, the discriminant analysis also demonstrated the overlap among isolates from different geographic regions, however, isolates from different hosts did cluster according to their hosts. The dendrogram construction based on Nei’s genetic distance using UPGMA for the isolates from 14 different geographic regions and three hosts also showed that isolates were grouped into three major clusters according to their hosts. The three host populations of *V. carpophila* showed significant differences in colony morphology, growth rate and spore production on the MEA medium. The cultures of *V. carpophila* from California almond trees and from peach trees in the southeastern United States showed similar results in which differences were observed for isolates from different hosts (Chen et al., 2014). This biological observation indicated that *V. carpophila* isolates from different hosts had significant differences, which were consistent with the results obtained by molecular analysis based on SSR markers. These differences might be caused by host specialization during the evolution of *V. carpophila*.

## Conclusion

In brief, the genetic diversity and population structure of the *V. carpophila* have rarely been characterized. This is the first report of the genetic variation and population structure in *V. carpophila* isolates from China. We demonstrated that Chinese *V. carpophila* populations could be clustered into three distinct genetic groups, which correspond to the host boundaries, i.e., peach, mume, and apricot. Based on peach isolates from 14 provinces, it is obvious that *V. carpophila* isolates do not group according to the geographic regions. These results show that the host but not the environment play an important role during the evolution of *V. carpophila.* In other words, the genetic identity of *V. carpophila* isolates is dependent on the host, but not the geographic region. This research laid a solid foundation for a better understanding of the population biology and genetics of *V. carpophila*. The global geographic populations and more host populations of *V. carpophila* should be further explored in the future.

## Data availability statement

The datasets presented in this study can be found in online repositories. The names of the repository/repositories and accession number(s) can be found in the article/Supplementary material.

## Author contributions

C-XL and YZ designed the study. YZ performed all the experiments. YZ, CC, Y-FW, and ML analyzed the data and drafted the manuscript. C-XL, YZ, FF, L-FY, and W-XY reviewed and edited the manuscript. All authors contributed to the article and approved the submitted version.

## Funding

This work was supported by the China Agriculture Research System of the Ministry of Finance and the Ministry of Agriculture and Rural Affairs (No. CARS30-3-03), the Fundamental Research Funds for the Central Universities (No. 2662020ZKPY018), Central Public-interest Scientific Institution Basal Research Fund (No. 1610172022011), and the National Natural Science Foundation of China (No.32202389).

## Conflict of interest

The authors declare that the research was conducted in the absence of any commercial or financial relationships that could be construed as a potential conflict of interest.

## Publisher’s note

All claims expressed in this article are solely those of the authors and do not necessarily represent those of their affiliated organizations, or those of the publisher, the editors and the reviewers. Any product that may be evaluated in this article, or claim that may be made by its manufacturer, is not guaranteed or endorsed by the publisher.

## References

[ref1] Bailly C. (2020). Anticancer properties of Prunus mume extracts (Chinese plum, Japanese apricot). J. Ethnopharmacol. 246:112215. doi: 10.1016/j.jep.2019.112215, PMID: 31491438

[ref2] Baroudy F. Putman A. I. Habib W. Puri K. D. Subbarao K. V. Nigro F. (2019). Genetic diversity of Verticillium dahliae populations from olive and potato in Lebanon. Plant Dis. 103, 656–667. doi: 10.1094/PDIS-03-18-0420-RE, PMID: 30823856

[ref3] Bock C. H. Thrall P. H. Burdon J. J. (2005). Genetic structure of populations of Alternaria brassicicola suggests the occurrence of sexual recombination. Mycol. Res. 109, 227–236. doi: 10.1017/S0953756204001674, PMID: 15839106

[ref4] Bock C. H. Wood B. W. Stevenson K. L. Arias R. S. (2014). Genetic diversity and population structure of Fusicladium effusum on pecan in the United States. Plant Dis. 98, 916–923. doi: 10.1094/PDIS-12-13-1229-RE, PMID: 30708843

[ref5] Bock C. Young C. Zhang M. Chen C. Brannen P. M. Adaskaveg J. E.. (2020). Mating type idiomorphs, heterothallism and high genetic diversity in Venturia carpophila, cause of peach scab. Phytopathology 111, 408–424. doi: 10.1094/PHYTO-12-19-0485-R, PMID: 32748736

[ref6] Bruvo R. Michiels N. K. D'Souza T. G. Schulenburg H. (2004). A simple method for the calculation of microsatellite genotype distances irrespective of ploidy level. Mol. Ecol. 13, 2101–2106. doi: 10.1111/j.1365-294X.2004.02209.x, PMID: 15189230

[ref7] Chen C. Bock C. H. Brannen P. M. Adaskaveg J. E. (2018). Mining and characterization of microsatellites from a genome of Venturia carpophila. Mycol. Prog. 17, 885–895. doi: 10.1007/s11557-018-1401-x

[ref8] Chen C. Bock C. H. Brannen P. M. Adaskaveg J. E. Hotchkiss M. W. Brewer M. T.. (2014). Genetic variability among populations of Fusicladium species from different host trees and geographic locations in the USA. Mycol. Prog. 13, 1179–1190. doi: 10.1007/s11557-014-1006-y

[ref9] Chen C. Bock C. H. Wood B. W. (2017). Draft genome sequence of Venturia carpophila, the causal agent of peach scab. Stand Genomic Sci. 12:68. doi: 10.1186/s40793-017-0280-0, PMID: 29213355 PMC5712196

[ref10] Chen S. Schnabel G. Yuan H. Luo C. (2019). LAMP detection of the genetic element 'Mona' associated with DMI resistance in Monilinia fructicola. Pest Manag. Sci. 75, 779–786. doi: 10.1002/ps.5178, PMID: 30125043

[ref11] Choi E. D. Kim G. H. Park S.-Y. Hoon Song J. Lee Y. S. Jung J. S.. (2019). Genetic diversity of the pear scab fungus Venturia nashicola in Korea. Mycobiology 47, 76–86. doi: 10.1080/12298093.2019.1572263, PMID: 31001451 PMC6452914

[ref12] Chuda Y. Ono H. Ohnishi-Kameyama M. Matsumoto K. Nagata T. Kikuchi Y. (1999). Mumefural, citric acid derivative improving blood fluidity from fruit-juice concentrate of Japanese apricot (Prunus mume Sieb. Et Zucc). J. Agric. Food Chem. 47, 828–831. doi: 10.1021/jf980960t, PMID: 10552374

[ref13] Dar M. Ahmad M. Shah M. U. D. Bhat N. Khan A. Padder B. A. (2019). Phylogenetic relationship of Venturia carpophila, the causal agent of almond scab from Kashmir valley as inferred by ITS nr DNA. Int. J. of Curr. Microbiol. Appl. Sci. 8, 2913–2919. doi: 10.20546/ijcmas.2019.806.350

[ref14] Earl D. A. vonHoldt B. M. (2012). Structure harvester: a website and program for visualizing structure output and implementing the evanno method. Conserv. Genet. Resour. 4, 359–361. doi: 10.1007/s12686-011-9548-7

[ref15] Ebrahimi L. Fotuhifar K.-B. Javan Nikkhah M. Naghavi M.-R. Baisakh N. (2016). Population genetic structure of apple scab (Venturia inaequalis (Cooke) G. winter) in Iran. PLoS One 11:e0160737. doi: 10.1371/journal.pone.0160737, PMID: 27631622 PMC5025049

[ref16] Evanno G. Regnaut S. Goudet J. (2005). Detecting the number of clusters of individuals using the software structure: a simulation study. Mol. Ecol. 14, 2611–2620. doi: 10.1111/j.1365-294X.2005.02553.x, PMID: 15969739

[ref17] Falush D. Stephens M. Pritchard J. K. (2007). Inference of population structure using multilocus genotype data: dominant markers and null alleles. Mol. Ecol. Notes 7, 574–578. doi: 10.1111/j.1471-8286.2007.01758.x, PMID: 18784791 PMC1974779

[ref18] Fan J. Y. Guo L. Y. Xu J. P. Luo Y. Michailides T. J. (2010). Genetic diversity of populations of Monilinia fructicola (fungi, ascomycota, helotiales) from China. J. Eukaryot. Microbiol. 57, 206–212. doi: 10.1111/j.1550-7408.2009.00467.x, PMID: 20113378

[ref19] Fisher E. E. (1961). Venturia carpophila sp.nov., the ascigerous state of the apricot freckle fungus. Trans. Br. Mycol. Soc. 44, 337–IN4. doi: 10.1016/S0007-1536(61)80026-0

[ref20] Gladieux P. Zhang X. G. Afoufa-Bastien D. Valdebenito Sanhueza R. M. Sbaghi M. Le Cam B. (2008). On the origin and spread of the scab disease of apple: out of Central Asia. PLoS One 3:e1455. doi: 10.1371/journal.pone.0001455, PMID: 18197265 PMC2186383

[ref21] González-Domínguez E. Armengol J. Rossi V. (2017). Biology and epidemiology of Venturia species affecting fruit crops: a review. Front. Plant Sci. 8:1496. doi: 10.3389/fpls.2017.01496, PMID: 28974954 PMC5610699

[ref22] Guérin F. Franck P. Loiseau A. Devaux M. Le Cam B. (2004). Isolation of 21 new polymorphic microsatellite loci in the phytopathogenic fungus Venturia inaequalis. Mol. Ecol. Notes 4, 268–270. doi: 10.1111/j.1471-8286.2004.00637.x

[ref23] He R. Liu P. Jia B. Xue S. Wang X. Hu J.. (2019). Genetic diversity of pseudomonas syringae pv. Actinidiae strains from different geographic regions in China. Phytopathology 109, 347–357. doi: 10.1094/PHYTO-06-18-0188-R, PMID: 30226424

[ref24] He Z. Zhang H. Gao S. Lercher M. J. Chen W. H. Hu S. (2016). Evolview v2: an online visualization and management tool for customized and annotated phylogenetic trees. Nucleic Acids Res. 44, 236–241. doi: 10.1093/nar/gkw370 PMC4987921 27131786

[ref25] Jiménez-Díaz R. M. Olivares-García C. Landa B. B. del Mar Jiménez-Gasco M. Navas-Cortés J. A. (2011). Region-wide analysis of genetic diversity in Verticillium dahliae populations infecting olive in southern Spain and agricultural factors influencing the distribution and prevalence of vegetative compatibility groups and pathotypes. Phytopathology 101, 304–315. doi: 10.1094/PHYTO-07-10-0176, PMID: 20942654

[ref26] Jombart T. Devillard S. Balloux F. (2010). Discriminant analysis of principal components: a new method for the analysis of genetically structured populations. BMC Genet. 11:94. doi: 10.1186/1471-2156-11-94, PMID: 20950446 PMC2973851

[ref27] Kim G. H. Jo K. Y. Shin J. S. Shin G. H. Koh Y. J. (2017). Epidemiological characteristics of scab of Japanese apricot in Korea. Plant Pathol. J. 33, 450–457. doi: 10.5423/PPJ.OA.03.2017.0044, PMID: 29018308 PMC5624487

[ref28] Koh H. S. Sohn S. H. Lee Y. S. Koh Y. J. Song J. H. Jung J. S. (2013). Specific and sensitive detection of Venturia nashicola, the scab fungus of Asian pears, by nested PCR. Plant Pathol. J. 29, 357–363. doi: 10.5423/PPJ.OA.06.2013.0055, PMID: 25288964 PMC4174815

[ref29] Leroy T. Lemaire C. Dunemann F. Le C. B. (2013). The genetic structure of a Venturia inaequalis population in a heterogeneous host population composed of different malus species. BMC Evol. Biol. 13:64. doi: 10.1186/1471-2148-13-64, PMID: 23497223 PMC3626921

[ref30] Li M. Zheng P. Ni B. Hu X. Miao X. Zhao Z. (2018). Genetic diversity analysis of apricot cultivars grown in China based on SSR markers. Eur. J. Hortic. 83, 18–27. doi: 10.17660/eJHS.2018/83.1.3

[ref31] Padder B. A. Sofi T. A. Ahmad M. Shah M.-U.-D. Hamid A. Saleem S.. (2013). Virulence and molecular diversity of Venturia inaequalis in commercial apple growing regions in Kashmir. J. Phytopathol. 161, 271–279. doi: 10.1111/jph.12061

[ref32] Peakall R. Smouse P. E. (2006). Genalex 6: genetic analysis in excel. Population genetic software for teaching and research. Mol. Ecol. Notes 6, 288–295. doi: 10.1111/j.1471-8286.2005.01155.x PMC3463245 22820204

[ref33] Poniatowska A. Michalecka M. Puławska J. (2016). Genetic diversity and pathogenicity of Monilinia polystroma the new pathogen of cherries. Plant Pathol. 65, 723–733. doi: 10.1111/ppa.12463

[ref34] Scherm H. Savelle A. T. Boozer R. T. Foshee W. G. (2008). Seasonal dynamics of conidial production potential of Fusicladium carpophilum on twig lesions in southeastern peach orchards. Plant Dis. 92, 47–50. doi: 10.1094/PDIS-92-1-0047, PMID: 30786365

[ref35] Schnabel G. Layne D. R. (2004). Comparison of reduced-application and sulfur-based fungicide programs on scab intensity, fruit quality, and cost of disease control on peach. Plant Dis. 88, 162–166. doi: 10.1094/PDIS.2004.88.2.162, PMID: 30812423

[ref36] Shen M. Zhang J. Q. Zhao L. L. Groenewald J. Z. Crous P. W. Zhang Y. (2020). Venturiales. Stud. Mycol. 96, 185–308. doi: 10.1016/j.simyco.2020.03.001, PMID: 32904190 PMC7452091

[ref37] Singh J. Khan A. (2019). Distinct patterns of natural selection determine sub-population structure in the fire blight pathogen, Erwinia amylovora. Sci. Rep. 9:14017. doi: 10.1038/s41598-019-50589-z, PMID: 31570749 PMC6768868

[ref38] Sitther V. Garrido Haro P. A. Molineros J. E. Garzon C. D. Jiménez-Gasco M. M. (2018). Genetic diversity of apple-and crabapple-infecting isolates of Venturia inaequalis in Pennsylvania, the United States, determined by microsatellite markers. For. Path. 48:e12405. doi: 10.1111/efp.12405

[ref39] Sivanesan A. (1977). The taxonomy and pathology of Venturia species. Bibl. Mycol. 59:139.

[ref40] Sun X. Kang S. Zhang Y. Tan X. Yu Y. He H.. (2013). Genetic diversity and population structure of rice pathogen Ustilaginoidea virens in China. PLoS One 8:e76879. doi: 10.1371/journal.pone.0076879, PMID: 24098811 PMC3786968

[ref41] Tenzer I. Gessler C. (1999). Genetic diversity of Venturia inaequalis across Europe. Eur. J. Plant Pathol. 105, 545–552. doi: 10.1023/A:1008775900736

[ref42] Turan C. Nanni I. M. Brunelli A. Collina M. (2015). New rapid DNA extraction method with Chelex from Venturia inaequalis spores. J. Microbiol. Methods 115, 139–143. doi: 10.1016/j.mimet.2015.06.005, PMID: 26079986

[ref43] Vilgalys R. Hester M. (1990). Rapid genetic identification and mapping of enzymatically amplified ribosomal DNA from several Cryptococcus species. J. Bacteriol. 172, 4238–4246. doi: 10.1128/jb.172.8.4238-4246.1990, PMID: 2376561 PMC213247

[ref44] Villarino M. Larena I. Martinez F. Melgarejo P. De Cal A. (2012). Analysis of genetic diversity in Monilinia fructicola from the Ebro valley in Spain using ISSR and RAPD markers. Eur. J. Plant Pathol. 132, 511–524. doi: 10.1007/s10658-011-9895-y

[ref45] White T. J. Bruns T. Lee S. Taylor J. (1990). “Amplification and direct sequencing of fungal ribosomal RNA genes for phylogenetics” in PCR protocols, a guide to methods and applications. Eds. M. A. Innis, D. H. Gelfand, J. J. Sninsky, and T. J. White (San Diego: Academic), 315–322.

[ref46] Yeh F. C. Yahg R. Boyle T. J. Ye Z. Xiyan J. M. (1999). POPGENE 32, Microsoft windows-based freeware for population genetic analysis, version 1.32, Molecular Biology and Biotechnology Centre. Edmonton, Canada: University of Alberta. http://www.ualberta.ca/~fyeh/popgene_download.html.

[ref47] Yu M. Yu J. Li H. Wang Y. Yin X. Bo H.. (2016). Survey and analysis of simple sequence repeats in the Ustilaginoidea virens genome and the development of microsatellite markers. Gene 585, 28–34. doi: 10.1016/j.gene.2016.03.016, PMID: 26992636

[ref48] Zhou Y. Fan F. Chaisiri C. Zhu Y. T. Zhao Y. Luo M. K.. (2021a). Sensitivity of Venturia carpophila from China to five fungicides and characterization of carbendazim-resistant isolates. Plant Dis. 105, 3990–3997. doi: 10.1094/PDIS-04-21-0694-RE, PMID: 34253040

[ref49] Zhou Y. Fan F. Wang L. Chaisiri C. Yin L. F. Yin W. X.. (2020). Development of a loop-mediated isothermal amplification method for the rapid detection of Venturia carpophila on peach. Pest Manag. Sci. 77, 1383–1391. doi: 10.1002/ps.6154, PMID: 33098187

[ref50] Zhou Y. Zhang L. Fan F. Wang Z. Q. Huang Y. Yin L. F.. (2021b). Genome sequence of Venturia carpophila, the causal agent of peach scab. Mol. Plant Microbe 34, 852–856. doi: 10.1094/MPMI-11-20-0321-A, PMID: 33656373

[ref51] Zhou Y. Zhang L. Ji C. Y. Chaisiri C. Yin L. F. Yin W. X.. (2022). Cytological observation of the infectious process of Venturia carpophila on peach leaves. Plant Dis. 106, 79–86. doi: 10.1094/PDIS-03-21-0556-RE, PMID: 34433321

