# Supplementary material for: Genetic diversity of Venturia carpophila populations from different hosts and geographic regions in China
Source: Front Microbiol. 2022 Dec 16;13:985691. doi: 10.3389/fmicb.2022.985691 (PMC9800423; doi:10.3389/fmicb.2022.985691)
Supplement: Supplementary file 1 [file Data_Sheet_1.doc]

**10 20 30 40 50 60 70 80 90**

***....|....| ....|....| ....|....| ....|....| ....|....| ....|....| ....|....| ....|....| ....|....|***

**MN958568.1**  **GTCGTAACAA** **GGTTTCCGTA** **GGTGAACCTG** **CGGAAGGATC** **ATTAATGGGT** **TTCCCGCCTC** **CGGGCGCAAC** **TCCACCCTTT** **GCAACCGCGG**

**MN958609.1**  **..........** **..........** **..........** **..........** **..........** **..........** **..........** **..........** **..........**

**MN958640.1**  **..........** **..........** **..........** **..........** **..........** **..........** **..........** **..........** **..........**

**MN958584.1**  **..........** **..........** **..........** **..........** **..........** **..........** **..........** **..........** **..........**

**MN958592.1**  **..........** **..........** **..........** **..........** **..........** **..........** **..........** **..........** **..........**

**100 110 120 130 140 150 160 170 180**

***....|....| ....|....| ....|....| ....|....| ....|....| ....|....| ....|....| ....|....| ....|....|***

**MN958568.1**  **CCCGGATTCG** **GCGCCTGGCG** **AGGACCACCC** **CCCAGACGGG** **GGGCCCCGCC** **TGCCGGAATC** **AGCAAGCCCT** **GCCTAGAAAA** **TTGAAGTCTG**

**MN958609.1**  **..........** **..........** **..........** **..........** **..........** **..........** **..........** **..........** **..........**

**MN958640.1**  **..........** **..........** **G.........** **..........** **..........** **..........** **..........** **..........** **..........**

**MN958584.1**  **..........** **..........** **G.........** **..........** **..........** **..........** **..........** **..........** **..........**

**MN958592.1**  **..........** **..........** **..........** **..........** **..........** **..........** **..........** **..........** **..........**

**190 200 210 220 230 240 250 260 270**

***....|....| ....|....| ....|....| ....|....| ....|....| ....|....| ....|....| ....|....| ....|....|***

**MN958568.1**  **AGGAGAAAGC** **CAAACGAAAA** **AAACTTTCAA** **CAACGGATCT** **CTTGGTTCTG** **GCAACGATGA** **AGAACGCAGC** **GAAATGCGAT** **AAGTAATGTG**

**MN958609.1**  **..........** **..........** **..........** **..........** **..........** **..........** **..........** **..........** **..........**

**MN958640.1**  **..........** **..........** **..........** **..........** **..........** **..........** **..........** **..........** **..........**

**MN958584.1**  **..........** **..........** **..........** **..........** **..........** **..........** **..........** **..........** **..........**

**MN958592.1**  **..........** **..........** **..........** **..........** **..........** **..........** **..........** **..........** **.......A..**

**280 290 300 310 320 330 340 350 360**

***....|....| ....|....| ....|....| ....|....| ....|....| ....|....| ....|....| ....|....| ....|....|***

**MN958568.1**  **AATTGCAGAA** **TTCAGTGAAT** **CATCGAATCT** **TTGAACGCAC** **ATTGCGCCCC** **CTGGTATTCC** **GGGGGGCACG** **CCTGTTCGAG** **CGCCATTTCT**

**MN958609.1**  **..........** **..........** **..........** **..........** **..........** **..........** **..........** **..........** **..........**

**MN958640.1**  **..........** **..........** **..........** **..........** **..........** **..........** **..........** **..........** **..........**

**MN958584.1**  **..........** **..........** **..........** **..........** **..........** **..........** **..........** **..........** **..........**

**MN958592.1**  **..........** **..........** **..........** **..........** **..........** **..........** **..........** **..........** **..........**

**370 380 390 400 410 420 430 440 450**

***....|....| ....|....| ....|....| ....|....| ....|....| ....|....| ....|....| ....|....| ....|....|***

**MN958568.1**  **ACCCTGGAGC** **CCCGCTCTGT** **GATGGGCCCC** **GTCCTCGCGG** **ACGAGCCCGA** **AACCCGTAGG** **CGCCGTCGCC** **CGGCCCCGAG** **CGTAGCAAGA**

**MN958609.1**  **..........** **..........** **..........** **..........** **..........** **..........** **..........** **..........** **..........**

**MN958640.1**  **..........** **..........** **..........** **..........** **..........** **..........** **..........** **..........** **..........**

**MN958584.1**  **..........** **..........** **..........** **..........** **..........** **..........** **..........** **..........** **..........**

**MN958592.1**  **..........** **..........** **..........** **..........** **..........** **..........** **..........** **..........** **..........**

**460 470 480 490 500 510 520 530 540**

***....|....| ....|....| ....|....| ....|....| ....|....| ....|....| ....|....| ....|....| ....|....|***

**MN958568.1**  **GAAATCCCTC** **GCCCGGAGCG** **CCCGGCGGTG** **GCCGCCCCGA** **AACCCTTCCA** **CAAGGTTGGC** **CTCGGATCAG** **GTGGGGATAC** **CCGCTGAACT**

**MN958609.1**  **..........** **..........** **..........** **..........** **..........** **..........** **..........** **..........** **..........**

**MN958640.1**  **..........** **..........** **..A.......** **..........** **..........** **..........** **..........** **..........** **..........**

**MN958584.1**  **..........** **..........** **..........** **..........** **..........** **..........** **..........** **..........** **..........**

**MN958592.1**  **..........** **..........** **..........** **..........** **..........** **..........** **..........** **..........** **..........**

**550**

***....|....| ....|***

**MN958568.1**  **TAAGCATATC** **AATAA**

**MN958609.1**  **..........** **.....**

**MN958640.1**  **..........** **.....**

**MN958584.1**  **..........** **.....**

**MN958592.1**  **..........** **.....**

**Fig. S1.** Based on ITS sequences, four haplotypes with one to two nucleotide changes were observed.


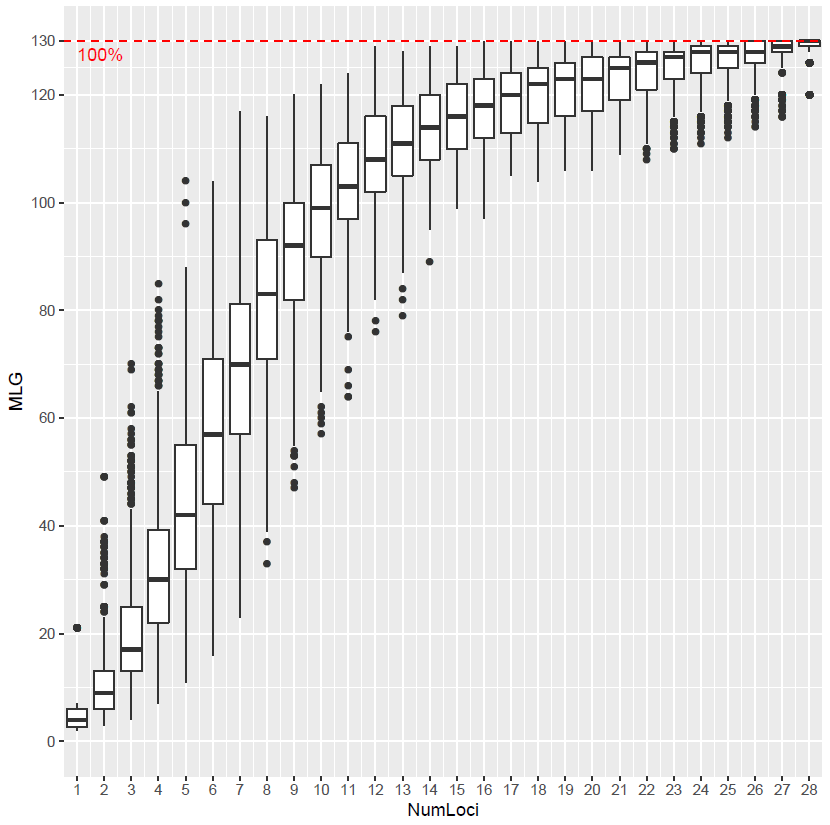


**Fig. S2.** Genotype accumulation curve of 186 *Venturia carpophila* isolates based on 29 SSR markers to determine the minimum number of microsatellite loci markers needed to distinguish isolates in a population.

| **Table S1. Number of alleles and PIC value for 31 simple sequence repeat (SSR) loci in 186 *Venturia carpophila* isolates sampled from China** | | | | |
| --- | --- | --- | --- | --- |
| Primer set | Forward primer sequence | Reverse primer sequence | N^a^ | PIC^b^ |
| Vc007 | ACGTTATGAGCAGTAGGTAGGGAG | AGGAAGTCGGTCATGAGAAAGAAC | 3 | 0.51 |
| Vc011 | AGGAGTAGTCGTACTTAAACCCCC | AGAGAAAGATTCGCTAATACCGC | 3 | 0.18 |
| Vc012 | AAAGATATTGGGTTCACATGCTCT | AAAAGTGAGAGATGGAAGGATGAG | 6 | 0.63 |
| Vc014 | AGAATTTCCCGTATGACTTGATGT | CAAGTCGATCTTGAGCATAGCATA | 2 | 0.04 |
| **Vc015** | AGAACAACCTCAAGGAGTAGCAGT | AGGTAGGTAGCAGTACCAGTCGAT | **1** | **0** |
| **Vc021** | GTAAGAAGCCTTACCCACTGTCTT | GCTTGAAGAATAGCAGTGTTGTGT | **1** | **0** |
| Vc026 | GGTAGTTCTGACGAGGTAGGATGT | ATTTCTGCACCTCCTTTAATTACG | 5 | 0.47 |
| Vc031 | ACTAAAGTGACCTGACAGCACAAC | CGACTACTGACTACTGCTGTCCTT | 3 | 0.22 |
| Vc038 | ATAGTCGGAGCAAATCTAGTGTCC | GACCTAGTCCAGAACACCAATGAT | 6 | 0.46 |
| Vc040 | GATGCTGATGATGATGAAGATGAT | GTCGTAGAGACGGTATGCACTATG | 7 | 0.74 |
| Vc043 | GAACTGAGATGGGAATGAAAAGTT | CCACCTGGAGCAAGTACAAGTAT | 2 | 0.50 |
| Vc044 | GTATACAGCACTTTTCAGGGTCAG | AGAAAGGAGGTAGGACCCTTGTT | 2 | 0.50 |
| Vc046 | TTATTCTTTATAGCTAGCCGTCCG | AGTGATTGATTCCATTCTTGTTGA | 7 | 0.64 |
| Vc055 | ATAGAGGCTTATTCCTTTCCATCC | TCAAAATGTCCCTTTTCCTTTATC | 4 | 0.55 |
| Vc056 | CAATCTCTACTACGATGACGGCT | AACAGTAGCTTAGTTGTCCCTTGG | 4 | 0.54 |
| Vc060 | ACGTTCGTAATAATAGGGAGCGTA | AACCTACGAAGGTACGAGTTTACC | 2 | 0.50 |
| Vc061 | GATGTGAGATAGCGGTAAGCAAG | AATCTCAACATCAAAGCTGCAAT | 2 | 0.08 |
| Vc077 | CCCACCTAGGACTGCTTAGTACAT | AAGAGATCGATCAAAGGAAATACG | 3 | 0.52 |
| Vc084 | GTCTTTGCTTGGGATTTCATTTT | GGTTCCACGTAGTAGCTAAGCATT | 3 | 0.15 |
| Vc085 | GGTAGGTAGGTACATGAAAGCCAC | GAAATGTGAAGTGTTGTCTCCATC | 3 | 0.63 |
| Vc087 | GAGTTTGACTTTGGTGTTTGTTTG | ATATCGAAGAAGAAAGAAGAGCGA | 6 | 0.60 |
| Vc089 | GTATCCTTTCTCTTCACCAATGCT | ACTTGAGAGTTCCTTGCTTCTTGT | 4 | 0.59 |
| **Vc090** | AAAGGAATAGAGTAGGAGGGTAATCTT | AGCCGTAGGGGTAGTAATAGAGGT | **0** | **0** |
| Vc097 | CATGATATTCTTGTCTGCGATACC | AGAACTTTCTGTCCCTGCTCTTC | 3 | 0.50 |
| Vc098 | GCGTATTTCGTAAATGCTCTATCC | TTTCAAGTTGTTTATGAGTGCAGA | 7 | 0.77 |
| Vc099 | CTACTGTGCAGAAACAAACATGC | TACCTATATATATCTCCGCGACCG | 3 | 0.18 |
| Vc106 | ACCTTCATCAAACAATCTTCATCC | GCAAGATTGACTTGACTTGACTTG | 2 | 0.03 |
| Vc109 | GTTATACCCAGATGGAATCCAGAC | GAAGAGAATGGAGGACAAGGG | 7 | 0.62 |
| Vc110 | GTATATACGTACGCAAGCCTACCC | GAAGCTAGAACGGGAGGAAAGT | 21 | 0.77 |
| Vc113 | AGTAAGAGCTGGGTGGTGTGAT | CCTTCGTTTCTGACCTACCATTAT | 6 | 0.53 |
| Vc118 | ATCGTTTTTCAGATCGGGGT | AATCCAAGTACCAAACCACCTAAA | 5 | 0.45 |
| Vc145 | CTTATGTCAGGTCCTGGAGACAC | TAATCATTCCCTAACGAAAGTTCC | 4 | 0.62 |

^a^ N= number of different alleles

^b^ PIC = 1−∑(*Pi*)^2^ (where *Pi* is the proportion of samples carrying the *i*th allele of a particular locus)

| **Table S2. Genetic diversity of *Venturia carpophila* on peach from 14 different provinces of China** | | | | | | |
| --- | --- | --- | --- | --- | --- | --- |
| Population | N* | *Na** | *Ne** | *H** | *I** | *PPL(NPL)** |
| Beijing (BJ) | 7 | 1.41±0.49 | 1.25±0.34 | 0.15±0.19 | 0.22±0.28 | 40.74%(55) |
| Chongqing (CQ) | 14 | 1.44±0.50 | 1.26±0.37 | 0.15±0.20 | 0.23±0.28 | 43.70%(59) |
| Guangdong (GD) | 6 | 1.19±0.40 | 1.19±0.40 | 0.10±0.20 | 0.13±0.27 | 19.26%(26) |
| Guangxi (GX) | 5 | 1.39±0.49 | 1.27±0.36 | 0.16±0.20 | 0.23±0.29 | 39.26%(53) |
| Guizhou (GZ) | 6 | 1.27±0.44 | 1.19±0.33 | 0.11±0.18 | 0.16±0.26 | 26.67%(36) |
| Hebei (HeB) | 6 | 1.16±0.37 | 1.13±0.30 | 0.07±0.16 | 0.10±0.24 | 16.30%(22) |
| Hubei (HuB) | 37 | 1.57±0.50 | 1.31±0.38 | 0.18±0.20 | 0.27±0.29 | 57.04%(77) |
| Henan (HN) | 5 | 1.36±0.48 | 1.27±0.38 | 0.15±0.21 | 0.22±0.30 | 36.30%(49) |
| Jiangsu (JS) | 7 | 1.41±0.49 | 1.29±0.38 | 0.17±0.21 | 0.24±0.30 | 41.48%(56) |
| Sichuan (SC) | 10 | 1.42±0.50 | 1.24±0.34 | 0.14±0.19 | 0.22±0.27 | 42.22%(57) |
| Shandong (SD) | 15 | 1.47±0.50 | 1.20±0.29 | 0.13±0.16 | 0.20±0.24 | 46.67%(63) |
| Shaanxi (SX) | 6 | 1.32±0.47 | 1.22±0.35 | 0.12±0.19 | 0.18±0.27 | 31.85%(43) |
| Yunnan (YN) | 5 | 1.29±0.45 | 1.18±0.31 | 0.11±0.18 | 0.16±0.26 | 28.86%(39) |
| Zhejiang (ZJ) | 27 | 1.50±0.50 | 1.28±0.39 | 0.16±0.20 | 0.24±0.28 | 50.37%(68) |
| Total | 156 | 1.78±0.42 | 1.32±0.39 | 0.18±0.20 | 0.28±0.28 | 77.78%(105) |

* N = No. of isolates

* *Na* = Observed number of alleles

* *Ne* = Effective number of alleles

* *H* = Nei's (1973) gene diversity

* *I* = Shannon's Information index

* *NPL* = Number of polymorphic loci

* *PPL* = Percentage of polymorphic loci

| **Table S3. Genetic diversity of *Venturia carpophila* from 3 different hosts at Huazhong Agricultural University (HZAU)** | | | | | | |
| --- | --- | --- | --- | --- | --- | --- |
| Population | N* | *Na** | *Ne** | *H** | *I** | *PPL(NPL)** |
| Peach (P) | 15 | 1.43±0.50 | 1.23±0.34 | 0.14±0.19 | 0.21±0.27 | 42.96%(58) |
| Mume (M) | 15 | 1.33±0.47 | 1.17±030 | 0.11±0.17 | 0.16±0.25 | 32.59%(44) |
| Apricot (A) | 15 | 1.57±0.50 | 1.28±0.33 | 0.17±0.18 | 0.26±0.26 | 57.04%(77) |
| Total | 45 | 1.76±0.43 | 1.34±0.34 | 0.21±0.18 | 0.33±0.25 | 75.56%(102) |

* N = No. of isolates

* *Na* = Observed number of alleles

* *Ne* = Effective number of alleles

* *H* = Nei's (1973) gene diversity

* *I* = Shannon's Information index

* *NPL* = Number of polymorphic loci

* *PPL* = Percentage of polymorphic loci

| **Table S4. Genetic similarity coefficients and genetic distances among *Venturia carpophila* isolates on peach collected from 14 province of China** | | | | | | | | | | | | | | |
| --- | --- | --- | --- | --- | --- | --- | --- | --- | --- | --- | --- | --- | --- | --- |
| Populations | Beijing | Chongqing | Guangdong | Guangxi | Guizhou | Hebei | Hubei | Henan | Jiangsu | Sichuan | Shandong | Shaanxi | Yunnan | Zhejiang |
| Beijing (BJ) | **** | 0.9744 | 0.934 | 0.9646 | 0.9376 | 0.9315 | 0.9717 | 0.9649 | 0.9717 | 0.9569 | 0.9394 | 0.9451 | 0.9383 | 0.9575 |
| Chongqing (CQ) | 0.0259 | **** | 0.9384 | 0.9691 | 0.9363 | 0.929 | 0.9799 | 0.9649 | 0.9709 | 0.9651 | 0.9341 | 0.9504 | 0.9414 | 0.9684 |
| Guangdong (GD) | 0.0682 | 0.0636 | **** | 0.9292 | **0.8819** | 0.9169 | 0.9378 | 0.9553 | 0.9433 | 0.9248 | 0.9270 | 0.9086 | 0.9114 | 0.9197 |
| Guangxi (GX) | 0.036 | 0.0314 | 0.0734 | **** | 0.9417 | 0.9512 | 0.9782 | 0.9552 | 0.9693 | 0.9435 | 0.9572 | 0.9492 | 0.9519 | 0.9702 |
| Guizhou (GZ) | 0.0645 | 0.0658 | **0.1257** | 0.0601 | **** | 0.9048 | 0.9511 | 0.9413 | 0.9333 | 0.9364 | 0.9237 | 0.9284 | 0.9252 | 0.9586 |
| Hebei (HeB) | 0.071 | 0.0736 | 0.0867 | 0.0500 | 0.1000 | **** | 0.9397 | 0.9519 | 0.9483 | 0.9297 | 0.9702 | 0.9055 | 0.8860 | 0.9251 |
| Hubei (HuB) | 0.0287 | 0.0203 | 0.0642 | 0.0220 | 0.0501 | 0.0622 | **** | 0.9648 | 0.9861 | 0.9491 | 0.9602 | 0.9394 | 0.9542 | **0.9882** |
| Henan (HN) | 0.0357 | 0.0357 | 0.0457 | 0.0458 | 0.0605 | 0.0493 | 0.0359 | **** | 0.9601 | 0.9707 | 0.963 | 0.9426 | 0.9356 | 0.9479 |
| Jiangsu (JS) | 0.0287 | 0.0295 | 0.0584 | 0.0312 | 0.069 | 0.0531 | 0.014 | 0.0407 | **** | 0.9431 | 0.9623 | 0.9277 | 0.9483 | 0.9747 |
| Sichuan (SC) | 0.0440 | 0.0355 | 0.0782 | 0.0582 | 0.0658 | 0.0729 | 0.0523 | 0.0298 | 0.0586 | **** | 0.9170 | 0.9418 | 0.9258 | 0.9372 |
| Shandong (SD) | 0.0625 | 0.0682 | 0.0758 | 0.0438 | 0.0794 | 0.0303 | 0.0406 | 0.0377 | 0.0384 | 0.0867 | **** | 0.9234 | 0.9204 | 0.9432 |
| Shaanxi (SX) | 0.0564 | 0.0509 | 0.0959 | 0.0521 | 0.0742 | 0.0993 | 0.0625 | 0.0592 | 0.0751 | 0.0599 | 0.0797 | **** | 0.9187 | 0.9271 |
| Yunnan (YN) | 0.0637 | 0.0603 | 0.0928 | 0.0493 | 0.0777 | 0.1211 | 0.0469 | 0.0666 | 0.0531 | 0.0771 | 0.0830 | 0.0848 | **** | 0.9396 |
| Zhejiang (ZJ) | 0.0434 | 0.0321 | 0.0837 | 0.0302 | 0.0423 | 0.0779 | **0.0118** | 0.0535 | 0.0256 | 0.0649 | 0.0585 | 0.0757 | 0.0623 | **** |

Genetic similarity coefficients (above diagonal) and genetic distance (below diagonal).

| **Table S5. Genetic similarity coefficients and genetic distances among *Venturia carpophila* isolates collected from 3 hosts in China** | | | |
| --- | --- | --- | --- |
| Populations | Peach (P) | Mume (M) | Apricot (A) |
| Peach (P) | **** | 0.8841 | 0.8742 |
| Mume (M) | 0.1231 | **** | **0.8616** |
| Apricot (A) | 0.1344 | **0.149** | **** |

Genetic similarity coefficients (above diagonal) and genetic distance (below diagonal).

| **Table S6.** **AMOVA of microsatellite data for *Venturia carpophila* isolates on peach grouped by geographic origins** | | | | | | | | | | |
| --- | --- | --- | --- | --- | --- | --- | --- | --- | --- | --- |
| Source | df | | | SS | MS | Est. Var. | Percentage | Stat | Value | *P* |
| Among provinces | | | 13 | 359.028 | 27.618 | 1.574 | **13%** | PhiPT | 0.125 | 0.001 |
| Within provinces | | 142 | | 1563.869 | 11.013 | 11.013 | 87% |  |  |  |
| Total | | 155 | | 1922.897 |  | 12.587 | 100% |  |  |  |

*df, degree of freedom; SS, sum of squared observations; MS, mean of squared observations; Est. Var., Estimated.Variance; PhiPT, proportion of the total genetic variance that are among individuals within a population.

| **Table S7.** **AMOVA of microsatellite data for *Venturia carpophila* isolates grouped by hosts (peach, mume and apricot)** | | | | | | | | |
| --- | --- | --- | --- | --- | --- | --- | --- | --- |
| Source | df | SS | MS | Est. Var. | Percentage | Stat | Value | P |
| Among host | 2 | 221.60 | 110.80 | 6.723 | **40%** | PhiPT | 0.403 | 0.001 |
| Within host | 42 | 418.40 | 9.962 | 9.962 | 60% |  |  |  |
| Total | 44 | 640.00 |  | 16.684 | 100% |  |  |  |

*df, degree of freedom; SS, sum of squared observations; MS, mean of squared observations; Est. Var., Estimated.Variance; PhiPT, proportion of the total genetic variance that are among individuals within a population.

| **Table S8. Pairwise comparisons of populations of *Venturia carpophila* on peach from different 14 provinces of China based on PhiPT values** | | | | | | | | | | | | | | |
| --- | --- | --- | --- | --- | --- | --- | --- | --- | --- | --- | --- | --- | --- | --- |
| Population | Beijing | Chongqing | Guangdong | Guangxi | Guizhou | Hebei | Hubei | Henan | Jiangsu | Sichuan | Shandong | Shaanxi | Yunnan | Zhejiang |
| Beijing (BJ) | **** | 0.001 | 0.001 | 0.001 | 0.001 | 0.001 | 0.001 | 0.001 | 0.001 | 0.001 | 0.001 | 0.001 | 0.001 | 0.001 |
| Chongqing (CQ) | 0.023 | **** | 0.001 | 0.001 | 0.001 | 0.001 | 0.001 | 0.001 | 0.001 | 0.001 | 0.001 | 0.001 | 0.001 | 0.001 |
| Guangdong (GD) | 0.196 | 0.194 | **** | 0.001 | 0.001 | 0.001 | 0.001 | 0.001 | 0.001 | 0.001 | 0.001 | 0.001 | 0.001 | 0.001 |
| Guangxi (GX) | 0.000 | 0.016 | 0.188 | **** | 0.001 | 0.001 | 0.001 | 0.001 | 0.001 | 0.001 | 0.001 | 0.001 | 0.001 | 0.001 |
| Guizhou (GZ) | 0.171 | 0.195 | 0.414 | 0.124 | **** | 0.001 | 0.001 | 0.001 | 0.001 | 0.001 | 0.001 | 0.001 | 0.001 | 0.001 |
| Hebei (HeB) | 0.236 | 0.247 | 0.369 | 0.127 | 0.392 | **** | 0.001 | 0.001 | 0.001 | 0.001 | 0.001 | 0.001 | 0.001 | 0.001 |
| Hubei (HuB) | 0.000 | 0.012 | 0.112 | 0.000 | 0.138 | 0.117 | **** | 0.001 | 0.001 | 0.001 | 0.001 | 0.001 | 0.001 | 0.001 |
| Henan (HN) | 0.165 | 0.161 | 0.297 | 0.170 | 0.284 | 0.358 | 0.132 | **** | 0.001 | 0.001 | 0.001 | 0.001 | 0.001 | 0.001 |
| Jiangsu (JS) | 0.216 | 0.241 | 0.272 | 0.094 | 0.297 | 0.117 | 0.095 | 0.330 | **** | 0.001 | 0.001 | 0.001 | 0.001 | 0.001 |
| Sichuan (SC) | 0.107 | 0.165 | 0.228 | 0.031 | 0.196 | 0.039 | 0.045 | 0.237 | 0.000 | **** | 0.001 | 0.001 | 0.001 | 0.001 |
| Shandong (SD) | 0.030 | 0.055 | 0.195 | 0.000 | 0.092 | 0.230 | 0.034 | 0.140 | 0.154 | 0.094 | **** | 0.001 | 0.001 | 0.001 |
| Shaanxi (SX) | 0.074 | 0.060 | 0.236 | 0.000 | 0.086 | 0.189 | 0.010 | 0.243 | 0.096 | 0.066 | 0.022 | **** | 0.001 | 0.001 |
| Yunnan (YN) | 0.240 | 0.166 | 0.467 | 0.165 | 0.212 | **0.509** | 0.160 | 0.425 | 0.408 | 0.339 | 0.170 | 0.030 | **** | 0.001 |
| Zhejiang (ZJ) | 0.140 | 0.129 | 0.285 | 0.068 | 0.154 | 0.297 | 0.097 | 0.309 | 0.229 | 0.211 | 0.108 | 0.000 | 0.000 | **** |

| **Table S9. Pairwise comparisons of populations of *Venturia carpophila* from different hosts in China based on PhiPT values** | | | |
| --- | --- | --- | --- |
| Population | Peach (P) | Mume (M) | Apricot (A) |
| Peach (P) | **** | 0.001 | 0.001 |
| Mume (M) | 0.418 | **** | 0.001 |
| Apricot (A) | 0.367 | **0.426** | **** |
